# A histopathological classification scheme for abdominal aortic aneurysm disease

**DOI:** 10.1016/j.jvssci.2021.09.001

**Published:** 2021-10-07

**Authors:** Laura E. Bruijn, Charid G. van Stroe Gómez, John A. Curci, Jonathan Golledge, Jaap F. Hamming, Greg T. Jones, Regent Lee, Ljubica Matic, Connie van Rhijn, Patrick W. Vriens, Dick Wågsäter, Baohui Xu, Dai Yamanouchi, Jan H. Lindeman

**Affiliations:** a Division of Vascular Surgery, Department of Surgery, Leiden University Medical Center (LUMC), Leiden, the Netherlands; b Section of Surgical Sciences, Department of Vascular Surgery, Vanderbilt University Medical Center, Nashville, Tenn; c Queensland Research Centre for Peripheral Vascular Disease, College of Medicine and Dentistry, James Cook University, Townsville, Queensland, Australia; d Department of Vascular and Endovascular Surgery, The Townsville University Hospital, Townsville, Queensland, Australia; e Australian Institute of Tropical Health and Medicine, James Cook University, Townsville, Queensland, Australia; f Department of Surgical Sciences, Dunedin School of Medicine, University of Otago, Dunedin, New Zealand; g Nuffield Dept. of Surgical Sciences, University of Oxford, Headington, United Kingdom; h Department of Molecular Medicine and Surgery, Karolinska Institute, Stockholm, Sweden; i Department of Surgery, Elisabeth-TweeSteden Ziekenhuis, Tilburg, the Netherlands; j Department of Medical Cell Biology, Uppsala University, Uppsala, Sweden; k Department of Surgery, Division of Vascular Surgery, Stanford University School of Medicine, Stanford, Calif; l Department of Surgery, School of Medicine and Public Health, University of Wisconsin, Madison, Wisc

**Keywords:** Abdominal aortic aneurysm, Classification, Histology, Thoracic aortic aneurysm, Vascular remodeling

## Abstract

**Objective:**

Two consensus histopathological classifications for thoracic aortic aneurysms (TAAs) and inflammatory aortic diseases have been issued to facilitate clinical decision-making and inter-study comparison. However, these consensus classifications do not specifically encompass abdominal aortic aneurysms (AAAs). Given its high prevalence and the existing profound pathophysiologic knowledge gaps, extension of the consensus classification scheme to AAAs would be highly instrumental. The aim of this study was to test the applicability of, and if necessary to adapt, the issued consensus classification schemes for AAAs.

**Methods:**

Seventy-two AAA anterolateral wall samples were collected during elective and emergency open aneurysm repair performed between 2002 and 2013. Histologic analysis (hematoxylin and eosin and Movat Pentachrome) and (semi-quantitative and qualitative) grading were performed in order to map the histological aspects of AAA. Immunohistochemistry was performed for visualization of aspects of the adaptive and innate immune system, and for a more detailed analysis of atherosclerotic lesions in AAA.

**Results:**

Because the existing consensus classification schemes do not adequately capture the aspects of AAA disease, an AAA-specific 11-point histopathological consensus classification was devised. Systematic application of this classification indicated several universal features for AAA (eg, [almost] complete elastolysis), but considerable variation for other aspects (eg, inflammation and atherosclerotic lesions).

**Conclusions:**

This first multiparameter histopathological AAA consensus classification illustrates the sharp histological contrasts between thoracic and abdominal aneurysms. The value of the proposed scoring system for AAA disease is illustrated by its discriminatory capacity to identify samples from patients with a nonclassical (genetic) variant of AAA disease.

## Clinical Relevance

The pathophysiology of abdominal aortic aneurysm (AAA) disease remains an enigma. Histological evaluation is critical for appreciation of the tissue remodeling and spatial aspects of AAA disease. Histopathological classification schemes have been devised for aortic pathology. Unfortunately, these schemes do not specifically address the most common aortic pathology, AAA disease. In order to facilitate interstudy comparisons and pathophysiologic understanding of AAA disease, we here present a multiparameter consensus histopathological AAA classification scheme. The scheme clearly discriminates AAA disease from thoracic aortic aneurysm disease. Systematic implementation of this AAA classification system indicates a substantial biological variation for AAA disease, and stresses the need for adequate group sizes in order to adequately cover the natural variability. Examples illustrating the diagnostic value of the classification system are provided.

**Article Highlights**

• **Type of Research:** Human study
• **Key Findings:** Existing consensus classification schemes for aortic disease do not adequately capture the aspects of abdominal aortic aneurysl (AAA) disease. Therefore, an AAA-specific 11-point histopathological consensus classification was devised.
• **Take Home Message:** Systematic implementation of this AAA classification system indicates a substantial biological variation for AAA disease and stresses the need for adequate group sizes in order to adequately cover the natural variability of AAA disease.

Histopathological classification is crucial for aortic disease diagnosis and classification, and for a better understanding of aortic pathology. To facilitate clinical decision-making and interstudy comparisons, two consensus classifications with harmonized nomenclature and categorization for respectively thoracic aortic aneurysms (TAAs)^1^ and inflammatory aortic diseases^2^ have recently been issued. Notably, these consensus classifications do not specifically encompass abdominal aortic aneurysms (AAAs), by far the most prevalent form of aortic disease.

Given its high prevalence and the existing knowledge gaps, extension of the consensus classification scheme to AAA would be of major value. However, inclusion of AAA in the consensus classifications is potentially interfered by fundamental pathophysiologic differences between TAA and AAA.^3^, ^4^, ^5^ For instance, more than TAA, AAA disease comes with gross alternations of the vessel wall structure^5^ and associates with an extensive clinical^6^ and molecular variability,^7^ all of which may translate in a histopathological diversity. The goal of this study was to evaluate whether the existing aortic diseases consensus classification schemes can be applied to AAA. Because it was concluded that the schemes incompletely cover the histopathological spectrum of AAA disease, a first outline for classification system for AAA disease was devised.

## Methods

### Patients and tissue sampling

Materials used in this study were formalin-fixed and paraffin-embedded samples from the Leiden University Medical Center (LUMC) tissue bank that were stored at room temperature. Samples were collected either as control samples within clinical trials (LUMC Medical Ethical Review Board protocol numbers: P00.146; P06.019; P08.017), or under the former opt-out guidelines for ‘further use of human tissue’ of the Medical and Ethical Committee of the LUMC, The Netherlands. Sample collection and handling was performed in accordance with former code of conduct of the Dutch Federation of Biomedical Scientific Societies (current code available at: www.coreon.org). Removal of excess aneurysm wall is a standard procedure during open aneurysm repair. As such, this excess vessel wall material in the study was considered “waste” material by Dutch law, and, therefore exempted from ethical consent (WGBO Art. 7:467) when used in anonymously as is the case for the tissue bank.

This study was performed on 72 AAA anterolateral wall samples collected during elective (n = 45) and emergency (n = 27) open aneurysm repair performed between 2002 and 2013 in the LUMC or the Elisabeth-TweeSteden Ziekenhuis, the Netherlands. These samples were randomly selected from a patient number list of the vascular tissue bank from the Department of Vascular Surgery, Leiden, the Netherlands. This tissue bank contains more than 250 AAA samples. It was anticipated that this sample size would provide sufficient power to map the variation in morphological characteristics.

Considering the median age of 70 years of the patients with AAA, and the reported association between atherosclerotic disease and AAA disease, two types of reference samples were considered relevant: first, infrarenal aorta samples without signs of atherosclerotic disease derived from kidney grafts of young organ donors (details of these control samples have been previously described^8^). These controls represent the ‘normal’ aorta. The second set of references were infrarenal, non-aneurysmal aortic wall specimens from elderly (kidney) organ^8^ donors. These latter reference samples covered the full spectrum of advanced atherosclerotic disease (revised American Heart Association grading according to Virmani^9^): late fibroatheroma, thin cap fibroatheroma, and fibrocalcific plaque.

Aortic tissues were formalin-fixed, decalcified (Kristensen’s fluid), and paraffin-embedded. Quality control showed that long-term storage of the tissue blocks did not interfere with the (immuno) histochemical stainings. Blocks were serially sectioned (4 μm) and hematoxylin and eosin (H&E) and Movat’s Pentachrome stained. A subset (n = 5) of samples that had been split, which halves had either been or not been decalcified, was stained for Alizarin Red S. See Supplementary Protocol 1 (online only) for protocols. Images were captured by means of digital microscopy (Philips IntelliSite Pathology Solution Ultra-Fast Scanner, Eindhoven, the Netherlands).

The authors declare that all supporting data are available within the article and its supplementary files.

### Histological analysis

From each patient, one section was randomly chosen and used for histological grading. Histological hallmarks of AAA disease were first explored by two independent observers (L.E.B., C.S.G.) and the senior author (J.H.L.), and applied to the panel, whereafter validation was performed by experts in the field (J.A.C., J.G., J.F.H., G.T.J., R.L., L.M., P.W.V., D.W., B.X., D.Y.). Histological analysis was based on Movat Pentachrome and H&E stainings. Semi-quantitative grading was based on integration of the data for the full tissue section (average grade), whereas qualitative scores were based on the most severe aspect present.

Regional heterogeneity within AAA samples in the longitudinal axis was explored by analyzing grading the sequential tissue blocks from single patients (n = 4).

Immunohistochemistry (IHC) was performed on randomly chosen subsets of the sample set (n = 72). Details for the IHC protocols, including primary antibodies used ([Apolipoprotein B-100 (ApoB100); n = 5], T-cells [CD4/8; n = 20], B-cells [CD20; n = 20], mature B-cells [CD21; n = 4], macrophages/foam cells [CD68; n = 20], plasma cells [CD138; n = 4], chemotactic signal for B-cells [CXCL13; n = 4], erythrocyte membranes [Glycophorin-A; n = 5], neutrophils [Myeloperoxidase; n = 20], and mast cells [Tryptase; n = 20]) are provided in Supplementary Protocol 1 and Supplementary Table I (online only).^10^ Tonsil tissue was used as a positive control for inflammatory cells and CXCL13, whereas ApoB100 was validated on liver tissue.

### Statistical analysis

Analyses were performed with IBM SPSS Statistics 27.0 (Amsterdam, the Netherlands).

Cohen’s kappa coefficients (κ) were used to assess intra-observer repeatability.

Pearson correlation analysis was performed to explore associations between clinical and histological characteristics.

## Results

Aggregated patient characteristics of the 70 cases included in the analysis, 44 of elective and 26 of acute aneurysm repair, are summarized in Table I. Individual data, and the data for the two excluded cases is provided in Supplementary Table II (online only).

**Table I:** AAA patient characteristics (n = 70^a^)

	Elective (n = 44)	Acute (n = 26)
Male sex	77 (34)	85 (22)
Age, years	69 (67-74)	73 (66-77)
AAA diameter, mm	61 (54-71.8)	74.5 (64-90)
Smoker		
Current	45 (20)	39 (10)
Never	16 (7)	19 (5)
Former	39 (17)	42 (11)
Statin use	66 (29)	69 (18)
Antihypertensive medication use^b^	80 (35)	77 (20)
Diabetes	14 (6)	8 (2)
History of cerebrovascular accident	21 (9)	19 (5)
History of coronary atherosclerosis^c^	46 (20)	46 (12)
Atrial fibrillation	14 (6)	35 (9)
Peripheral arterial disease^d^		
Yes	27 (12)	19 (5)
No	43 (19)	39 (10
Unknown	30 (13)	42 (11)
COPD^e^		
Yes	25 (11)	19 (5)
No	25 (11)	19 (5)
Unknown	50 (22)	62 (16)

*AAA,* Abdominal aortic aneurysm; *COPD,* chronic obstructive pulmonary disease.

Data are presented as number (%) or median (interquartile range).

a Initially, 72 cases were included. Two cases were excluded from analysis, one Marfan patient and one with characteristics suggestive for a syndromatic AAA variant (multiple aortic branch vessel aneurysms and four second-degree family members with an AAA).

b Antihypertensive medications included angiotensin II receptor blockers, angiotensin-converting enzyme inhibitors, diuretics, and calcium channel blockers.

c Signs of coronary atherosclerosis were considered myocardial infarction and/or angina.

d Peripheral arterial disease was considered present based on intermittent claudication complemented by an abnormal ankle-brachial index (<0.9) or interventions such as percutaneous transluminal angioplasty.

e COPD was considered present based on symptoms and abnormal pulmonary function testing.

### Application of the existing consensus statements for classifying AAA

Upon systematic application of the “non-inflammatory” consensus classification scheme^1^ (Supplementary Table IIIa), it was concluded that this scheme only partially covers the pathological changes in AAA. More specifically, key aspects, such as mucoid extracellular matrix accumulation; elastic fiber fragmentation, thinning, and disorganization; laminar medial collapse and smooth muscle cell (SMC) disorganization,^1^ are either absent in AAA or are not adequately captured by the thresholds defined in the classification. In fact, although TAA is characterized by isolated medial fibrosis,^1^ AAA associates with comprehensive elastolysis, more pronounced SMC (nuclei) loss, and transmural fibrosis with full loss of the characteristic three-layered aortic wall structure (see Supplementary Figure 1.1, online only, for illustrations of the contrasts). SMC disorganization was observed in two AAA samples (Supplementary Figure 1.2, online only), which, in retrospect, were from a patient with Marfan syndrome and a patient with multiple aortic branch vessel aneurysms (iliac, femoral, and popliteal) and four second-degree family members with an AAA (Supplementary Table II, online only), suggestive for a non-classic, syndromic AAA variant. On basis of the distinct histological morphology and the clinical information, these samples were excluded from the analysis.

The “inflammatory” consensus classification scheme^2^ focused on inflammatory aorta diseases (ie, atherosclerosis, atherosclerosis with excessive inflammation [eg, “inflammatory atherosclerotic aneurysm”] and (peri)aortitis). AAA is not explicitly included as a separate entity, but is brought forward as complication of severe aortic atherosclerosis (“inflammatory atherosclerotic aneurysm”). The working group issued an initial classification scheme focusing on aortitis and peri-aortitis.^2^ In contrast to the semi-quantitative “non-inflammatory” consensus classification scheme,^1^ this “inflammatory” classification scheme^2^ is merely descriptive. Systematic evaluation of this “inflammatory” classification scheme^2^ (Supplemental Table IIIb, online only) showed that the initial scheme focusing on peri-aortitis and aortitis^2^ incompletely covered the AAA characteristics. To be more specific, although the inflammatory reaction observed in AAA^9^ could be subclassified as “mixed inflammatory pattern,”^2^ this generic term has limited discriminatory capacity, as it does not cover the profound and extensive remodeling that hallmarks AAA disease.

In the light of a suggested association between AAA and (severe) atherosclerosis in the consensus statement,^2^ systematic grading of the atherosclerotic lesions was performed in all 72 AAA samples included in this study. Although the evaluation confirmed presence of isolated atherosclerotic lesions in 97% (n = 68) of the cases, few of the lesions were classified as advanced. In fact, the most advanced lesions present were either classified as early (lipid pools^9^ [47%; n = 32]) or intermediate lesions (isolated necrotic cores^9^ [51%; n = 35]). Notably, clusters of intermediate/late stage lesions and dominance of stabilized fibrous calcified lesions,^9^ as observed in the non-aneurysmal aorta of the elderly controls,^11^ were absent in AAA. Clear contrasts were also observed for the spatial organization and distribution of the leukocyte subpopulations^10^ in AAA and aortic atherosclerosis (Supplementary Figure 1.3, online only). Consequently, it was concluded that convincing support for an assertion that an AAA is a variant of severe aortic atherosclerosis is lacking.

Based on the inventory, it was concluded that the two consensus classification schemes^1^^,^^2^ for aortic pathology fail to adequately appreciate the histopathological aspects and biological diversity of AAA disease.

### Mapping the histological spectrum of end-stage AAA disease

To adequately appreciate the histopathological variability of AAA disease, we performed an inventory of its (patho)histomorphological characteristics. Aspects emerging from this evaluation are incorporated in an 11-point semi-quantitative classification system that includes the following aspects: wall thickness, elastolysis, mesenchymal cell loss, fibrosis, neovascularization, adventitial adipogenic degeneration, calcification, lymphoid infiltration, lymphoid follicles, atherosclerotic lesions, and intraluminal thrombus organization. Individual aspects of the grading system are detailed in Table II (classification system), and illustrated in Supplementary Figs 2.1-2.9 (online only) (representative images illustrating the different categories in the classification).

**Table II:** Eleven-point histomorphological AAA classification

Histological aspect	Scoring		Main results	Commentary
Mesenchymal cell loss, % (pattern)	1	10-30	In 50% of AAA samples, the estimated mesenchymal cell loss exceeded 80%.	Cutoffs applied differ from the thresholds of “non-inflammatory” consensus classification,^1^ because the >30% lower limit of the most advanced group was inadequate to appreciate the differential extent of cell loss in AAA disease.
	2	30-80, patchy		
	3	30-80, diffuse		
	4	>80, patchy		
	5	>80, diffuse		
Pathological ECM remodeling				
Intimal/medial fibrosis, % (pattern)	*1*	10-30	(Sub) normal aortic wall structure with a clear intima-media border absent in all AAA samples.	Fibrosis grading in the ”non-inflammatory” classification^1^ focuses on a granular fibrotic pattern in the media as seen in TAA and was inadequate to cover the extent and pattern of fibrosis in AAA disease. Moreover, the media is indistinguishable from the intima due to fibrosis. Therefore, cutoffs were adapted and applied to the intimal-medial zones collectively.
	2	30-80, patchy		
	3	30-80, diffuse		
	4	>80, patchy		
	5	>80, diffuse		
Elastin	1	Remnants of elastin visible		All AAA samples were characterized by extreme elastolysis. If present, elastin was only observed as patchy remnants.
	2	No elastin visible		
Aorta wall thickness, mm	Q4	1.49-3.05	No clear association between wall thickness and rupture.	Included as a more quantitative matrix aspect, whereas mesenchymal cell loss and pathological ECM remodeling essentially capture qualitative AAA matrix aspects.
	Q3	1.10-1.48		
	Q2	0.80-1.09		
	Q1	0.41-0.79		
Inflammation				
A. Transmural lymphoid infiltrates	0	Low number of perivascular lymphocytes	Transmural unorganized infiltrations and lymphoid follicles were concurrently present in the majority (65%) of AAA. Follicles were mainly (65%) early TLO-like structures. T-cells were distributed transmurally, while B-cells were primarily located in adventitial lymphoid follicles (Supplementary Fig 7.1, online only). Indications were found for gross B-cell immaturity in TLO-like structures (Supplementary Fig 7.2, online only).	Inflammation was graded separately from atherosclerotic lesions, because of the distinctive inflammatory signature of AAA and aortic atherosclerosis (Supplementary Fig 5.3, online only). Two distinct nonexclusive patterns of lymphoid infiltrations were observed: A) diffuse, unorganized lymphoid infiltrations that were located in the intima, media, or adventitia. B) organized lymphoid follicles, exclusively located in a characteristic boundary zone (presumably reflects the former outer media), also called TLOs. Maturation of TLO-like structures were graded in analogy to the stages of secondary lymphoid follicles, as previously described for TLOs.^12^
	1	Small unorganized lymphoid infiltrates		
	2	Large unorganized lymphoid infiltrates		
B. TLO-like structures in adventitia	0	No lymphoid follicles		
	1	Early TLO-like structure		
	2	Late developed TLO-like structure		
Neovascularization	0	Into part 1	Neovascularization up to the ILT was observed.	Extension of small vessels in four equal zones is graded relative to the medio-adventitial border, in which they mainly originate.
	1	Into part 2		
	2	Into part 3		
	3	Into part 4		
Atherosclerotic lesions	0	None visible	Atherosclerotic lesions were present in 97% of AAA samples, of which mainly foam cells/lipid pools (46%) and necrotic cores (50%).	Although the location and morphologic characteristics of some necrotic cores differed from classic aortic atherosclerotic lesions (Supplementary 6, online only), lesion characteristics could well be graded on basis of morphologic aspects of the Virmani classification^9^ (Supplementary Fig 5.4, online only).
	1	Foam cells/lipid pool(s) directly under ILT		
	2	Foam cells/lipid pool(s) not directly under ILT		
	3	Necrotic core deeply embedded in aortic wall, without classic overlying fibrous cap		
	4	Superficial necrotic core, resembling classic atherosclerotic lesion		
	5	Calcified sheet, of so-called stabilized, fibrous calcified lesions.		
ILT organization	0	No ILT	Reorganizing ILTs were present in 28% of the AAA samples were and the majority of ILTs were organized (53%).	An adherent ILT is a common aspect in AAA, often with cholesterol crystals at the level of intimal border zone and different degrees of reorganization/maturation.
	1	Fibrin and/or cholesterol rich ILT, clearly delineated from intima		
	2	Reorganizing ILT: incorporation into intima (fading of clear demarcation zone between aneurysm wall and thrombus, including regions of intramural hemorrhage (s), no immune cell infiltration in ILT		
	3	Reorganizing ILT: incorporation into intima, immune cell infiltration in ILT		
	4	Organized: matrix deposition and spindle shaped cell (mesenchymal cells) ingrowth in ILT, with (out) calcifications		
	5	Highly organized: matrix deposition, spindle-shaped cell ingrowth and capillary ingrowth, with (out) calcifications		
Micro-calcifications	0	No micro-calcifications	Micro-calcifications in both the inner and outer wall are a universal AAA characteristic. Movat stainings are suitable for evaluation of calcification in decalcified sections. (Supplementary 9, online only).	In specimens with minimal calcification, calcification was confined to the ILT, associated with cholesterol crystals. Progressive micro-calcifications were positioned in the inner aortic wall (intraluminal 50% of the total intimal/medial zone) and/or the outer aortic wall (remaining 50% intimal/medial zone at the adventitial side). Macro-calcifications were present in necrotic cores or as calcified sheets, appreciated in *5.6 Atherosclerotic lesions*.
	1	In both inner and outer wall		
Adventitial adipogenic degeneration^a^	0	Absent	Common, but not universal AAA characteristic (96%)	Adipogenic degeneration was confined to the adventitia.
	1	Present		

*AAA,* Abdominal aortic aneurysm; *ECM,* extracellular matrix; *ILT,* intraluminal thrombus; *TLO*s, tertiary lymphoid organs.

a The appearance of infiltration of isolated adipocyte clusters in the adventitia, without connection of the periaortic adipose tissue, and with intertwining strands or matrix or immune cell infiltrates in between the clusters.^13^.

This classification system was applied on the 70 AAA samples, and results are summarized in a heat map (Table III). Excellent (>0.8) Cohen’s kappa coefficients were observed for all histological parameters (see top of Table III).

**Table III:** Visual representation (heat map) of histopathological heterogeneity of end-stage AAA disease

Patient No.	Fibrosis in intima/media zone (κ 0.80)	Mesenchymal cell loss in intima/media zone (κ 0.87)	Inflammation-transmural lymphoid infiltration (κ 0.88)	Inflammation-lymphoid follicles in adventitia (κ 0.93)	Atherosclerotic lesions (κ 0.89)	Wall thickness (intima + media)	Extent of neovessel formation (κ 0.92)	Intraluminal thrombus organization (κ 0.88)	Calcification (κ 0.99)	Elastic fiber degradation (κ 0.80)	Adventitial adipogenic degeneration (κ 1.0)
Elective											
1											
2											
3											
4											
5											
6											
7											
8											
9											
10											
11											
12											
13											
14											
15											
16											
17											
18											
19											
20											
21											
22											
23											
24											
25											
26											
27											
28											
29											
30											
31											
32											
33											
34											
35											
36											
37											
38											
39											
40											
41											
42											
43											
44											
45											
Acute-ruptured											
46											
47											
48											
49											
50											
51											
52											
53											
54											
55											
56											
57											
58											
59											
60											
61											
62											
63											
64											
65											
66											
67											
68											
Acute-symptomatic											
69											
70											
71											
72											

*AAA,* Abdominal aortic aneurysm.

For color legend, see Table II.

Every row represents an AAA wall sample (1-70). AAA samples are hierarchically clustered based on the most discriminative factors; respectively, the intima/media fibrosis content, mesenchymal cell loss, inflammation patterns, and atherosclerotic lesions.

For corresponding clinical characteristics per patient, see Supplemental Table II (online only).

**Fig 1:**
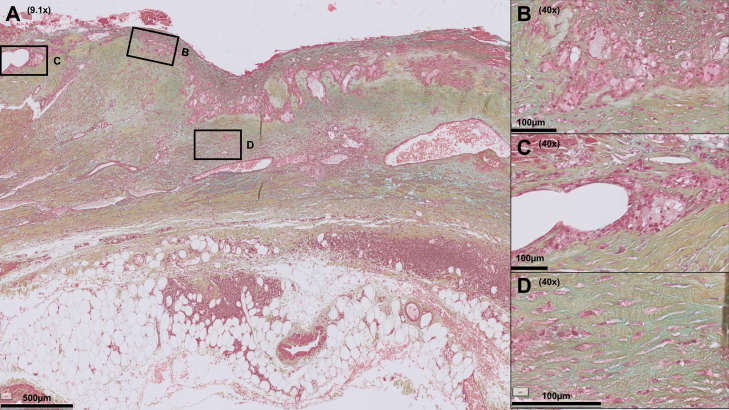
Abdominal aortic aneurysm (AAA) section showing high degree of inflammation and neovascularization, whereas the extracellular matrix is moderately fibrotic. In this AAA section, multiple inflammatory infiltrates are visible, note the lymphoid follicle (tertiary lymphoid organ-like structure) in the former media-adventitial border zone (**A**) and infiltrates of foam cells (**B**). Microvessels are visible up to the luminal side (**C**). The extracellular matrix is proteoglycan-rich (**D**; turquoise). Color legend of Movat Pentachrome staining: *blue*, proteoglycans; *yellow*, collagen; *green*, colocalization of proteoglycans and collagen; *black*, elastin; *red*, smooth muscle cells (SMC) and fibrinogen; *purple*, nuclei.

**Fig 2:**
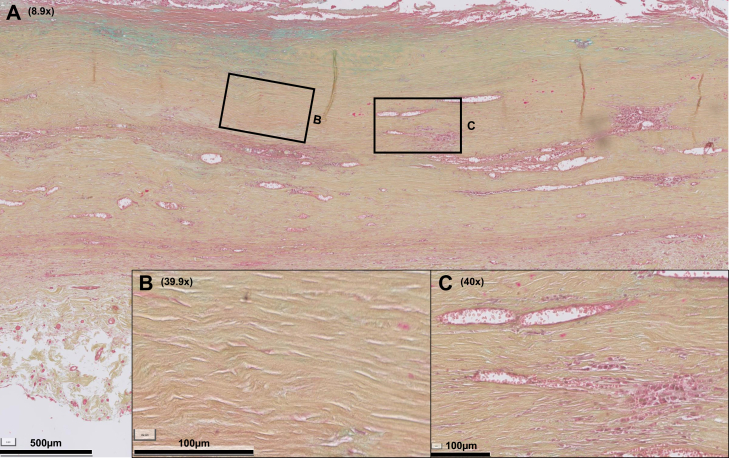
Abdominal aortic aneurysm (AAA) section showing high degree of fibrosis. **A,** This AAA section is characterized by extensive fibrotic changes (**B** for close-up): the extracellular matrix is rich in collagen, although poor in mesenchymal cells and elastin, reflected in the Movat staining by an ochre yellow cell matrix. The inflammation grade is relatively low, note multiple small perivascular infiltrates throughout the former intima/media zones (**C**). Color legend of Movat Pentachrome staining: *blue*, proteoglycans; *yellow*, collagen; *green*, colocalization of proteoglycans and collagen; *black*, elastin; *red*, smooth muscle cells (SMC) and fibrinogen; *purple*, nuclei.

**Fig 3:**
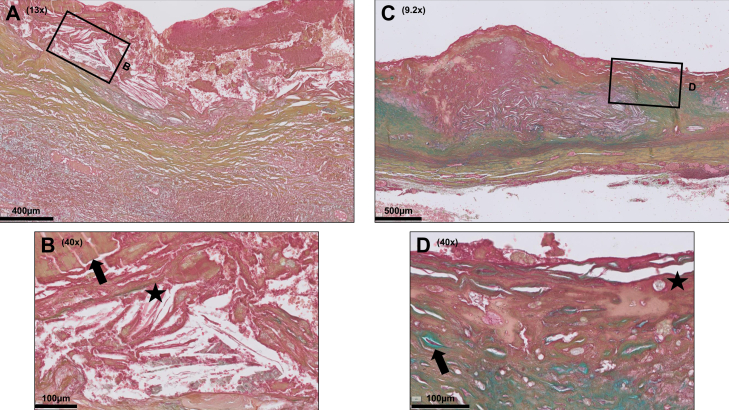
Intraluminal thrombi (ILT) in abdominal aortic aneurysm (AAA) sections show different grades of organization. An ILT is present in the majority of AAA disease. Various degrees of ILT reorganization were identified, ranging from relatively immature adhering ILTs (**A**) that are rich in fibrin (*arrow* in **B**) and cholesterol (*asterisk* in **B**; oblong spiked clefts) to highly organized ILTs, characterized by extracellular matrix deposition (**C**), spindle-shaped cell ingrowth (*arrow* in **D**), and ingrowth of microvessels (*asterisk* in **D**). Color legend of Movat Pentachrome staining: *blue*, proteoglycans; *yellow*, collagen; *green*, colocalization of proteoglycans and collagen; *black*, elastin; *red*, smooth muscle cells (SMC) and fibrinogen; *purple*, nuclei.

**Fig 4:**
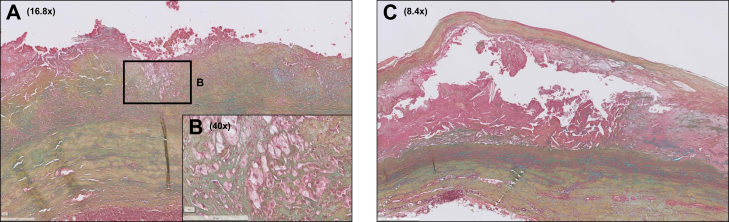
Atherosclerotic lesions in abdominal aortic aneurysm (AAA) sections range from minimal atherosclerosis to advanced atherosclerosis. The majority of AAA sections were characterized by minimal atherosclerosis (eg, infiltrates of foam cells on the luminal side as shown in insert **A**/**B**). However, progressive atherosclerotic lesions were also found, such as large necrotic cores on the luminal side (**C**). Color legend of Movat Pentachrome staining: *blue*, proteoglycans; *yellow*, collagen; *green*, colocalization of proteoglycans and collagen; *black*, elastin; *red*, smooth muscle cells (SMC) and fibrinogen; *purple*, nuclei.

From the systematic evaluation of the diverse histological aspects of AAA disease, the picture emerges of AAA being characterized by a series of consistent findings ([almost] complete elastolysis, micro-calcifications), as well as more varying factors (aortic wall thickness, fibrosis/mesenchymal cell loss, inflammation, atherosclerotic lesions, and the extent of neovascularization and intraluminal thrombus [ILT] organization). This histomorphological variability of AAA disease is illustrated in Fig 1, Fig 2, Fig 3, Fig 4.The scoring system did not identify specific signatures for ruptured and unruptured AAA samples (Table III).

A notable observation was the diffuse presence of cholesterol crystals at the transition zone of the ILT and the intima. To test whether the atherosclerotic lesions in AAA relate to lipoprotein accumulation or alternatively to erythrocyte membrane-derived cholesterol deposition, ApoB100 (lipoproteins) and Glycophorin-A (erythrocyte membrane) stainings were performed. Atherosclerotic lesions were all ApoB100-positive and Glycophorin-A-negative (Supplemental Figs 3.1 and 3.2, online only).

Specific stainings were also performed for a better characterization of tertiary lymphoid follicle-like structures in the adventitia and the diffuse, unorganized lymphoid infiltrates that were located in the intima, media, or adventitia. Tertiary lymphoid-follicle-like structures were identified on basis of the anatomical features (structures resembling lymph nodes, but that are located in locations that are normally devoid of canonical lymphoid organs, such as the aorta). It was concluded that these diffuse infiltrates mainly represented T-cells, and that the presence of B-cells and plasma cells was limited to the tertiary lymphoid follicle-like structures (Supplementary Fig 4.1, online only). These lymphoid follicles were positive for lymphoid homeostatic factor CXCL13, but negative for B-cell maturation marker CD21 (Supplementary Fig 4.2, online only).

Aspects of the adaptive immune cell signature in AAA disease are illustrated in Supplementary Fig 5 (online only). Macrophage infiltration was observed throughout all vessel walls, including in close proximity of the necrotic cores and within tertiary lymphoid follicle-like structures (Supplementary Fig 5.1, online only). Similarly, neutrophils and mast cells diffusely present throughout all vessel walls (Supplementary Fig 5.2 and 5.3, online only), where they primarily associated with microvessels (inner and medial wall segments) and or tertiary lymphoid follicle-like structures in the adventitial aspect of the aneurysm wall.

Exploration of regional variety within AAA samples from single patients indicated a modest variation in the core features (Supplementary Table IV, online only); the greatest variation was observed for atherosclerotic lesions.

Associations between clinical and discriminative histological parameters (viz. elastolysis, adipogenic adventitial degeneration, and microcalcifications were excluded) were explored by univariate Pearson correlation analysis (Supplementary Table V, online only). An inverse, robust association was found between aortic wall diameter and aortic wall thickness (R = 0.40; *P* < .05).

## Discussion

Although the consensus classifications schemes for aortic diseases^1^^,^^2^ prove highly instrumental for classifying TAA, it is concluded that the existing schemes fail to adequately cover the histological aspects of AAA disease. Therefore, a novel classification scheme is proposed to cover the unique characteristics and histological variability of AAA disease. Systematic application of this scheme highlighted extensive vascular remodeling as the universal core feature, but also pointed to an underappreciated heterogeneity for other key aspects (eg, inflammation, aspects of atherosclerosis, and ILT reorganization). No contrasts were observed between stable and ruptured AAA.

The issued histological consensus classification schemes for, respectively, inflammatory and non-inflammatory (degenerative) ascending aortic pathology are developed as standardized grading systems for aortic pathology.^1^^,^^2^ Given the parallels between TAA and AAA disease,^14^ and the prominent inflammatory aspects of AAA disease,^15^ we speculated that these classification systems could also be applied to AAA. However, following a systematic evaluation, it was concluded that aspects such as the profound transmural remodeling with extreme fibrosis and elastolysis, extensive mesenchymal cell loss, and completely distorted aortic wall structure in AAA disease are not adequately covered by the “non-inflammatory” classification scheme.^1^

AAAs are not included as a distinct entity in the “inflammatory” consensus scheme,^2^ although specific reference is made to “inflammatory aneurysms,” a type of aortic aneurysm that is clinically distinct from AAA, and that is characterized by wall thickening (>4 mm), extensive fibrosis, and extensive adhesions.^16^ The consensus scheme does refer to AAA as a complication of aortic atherosclerotic disease. A critical question is whether this assumption is justified. Epidemiologic and genetic evidence,^17^ contrasting histomorphologies of aortic atherosclerotic disease and AAA disease,^8^^,^^18^ and the atherosclerosis grading included in this evaluation classify AAA as a pathological entity that is distinct from atherosclerotic disease. In fact, although infrarenal aortas of an age-matched reference population are characterized by an intact three-layered vascular structure and abundant, often confluent end-stage fibrous calcified (stabilized) atherosclerotic lesions,^11^ atherosclerotic lesions in AAA disease are generally limited to isolated early (lipid pools) or intermediate (late fibroatheroma) lesions in the (former) intimal segment. Confluent fibrous-calcified lesions or their scars are notably rare in AAA. Altogether, these observations imply a reduced atherosclerosis burden in AAA disease; yet, it cannot be excluded that the characteristic scar-like FCP signatures of advanced atherosclerotic lesions in control aortas are lost in AAA disease as a consequence of the extensive vascular remodeling.

An apparently counterintuitive observation to the reduced atherosclerosis burden is the clusters of isolated cholesterol clefts that often present at the abluminal border zone of the thrombus. It could be speculated that these clefts reflect a cholesterol flux from membrane remnants of erythrocytes that caught in the thrombus. Erythrocyte membrane remnants have been recognized as a dominant cholesterol source in coronary atherosclerosis,^19^ and it was reasoned that such a mechanism could drive atherosclerotic lesion formation in AAA. ApoB100 and Glycophorin-A staining was performed to respectively discriminate between lipoprotein or erythrocyte membrane-driven atherosclerotic lesion formation in AAA disease. Consistent ApoB100 positivity but Glycophorin-A negativity suggests that the cholesterol fluxes in atherosclerotic lesions in AAA are essentially lipoprotein-driven.

Aspects of inflammation are obviously a core aspect of the “inflammatory” consensus scheme.^2^ In the consensus scheme, inflammation is grossly divided into two main categories: atherosclerosis (including inflammatory [atherosclerotic] aneurysms), and noninfectious and infectious (peri)aortitis. The question arises whether AAA aligns with one of the two main categories. The inflammatory signature of AAA is clearly distinct from atherosclerotic disease.^20^^,^^21^ For example, abundant adventitial follicles, a key histological feature of AAA disease, are rare in the context of atherosclerotic disease and in fact only, transiently, present in direct association with a thin cap or instable lesion type.^22^ Moreover, although immune aspects are clearly present in the AAA wall, absence of systemic involvement and the extensive vascular remodeling classify AAA as an entity that is clearly distinct from (peri)aortitis included in the consensus classification.

Because the “non-inflammatory” and “inflammatory” consensus classifications^1^^,^^2^ fail to cover the characteristics of AAA disease, a novel and unique AAA-specific classification scheme was devised based on a systematic histological inventory of 72 AAA samples. This first inventory resulted in an 11-point classification scheme that covers diverse histomorphological key aspects of AAA, some of which had been brought forward earlier as a histological characteristic of AAA disease.^23^^,^^24^ The inventory identified the extensive matrix remodeling ([near] complete loss of elastin, transmural fibrosis, and loss of the distinct boundaries between the three structural layers of the aorta) as the universal core features of AAA disease. In fact, re-evaluation of the patient records of two cases that presented with mild vascular remodeling implied that these aneurysms were not classical AAAs, but that they were part of a general dilating vascular disease (respectively, Marfan disease and an unknown familial trait). The pattern of matrix remodeling observed clearly differentiates AAA from TAA disease, which associates with a more localized, mucoid (glycoprotein rich) matrix remodeling and focal elastolysis.

Further histological aspects of AAA disease include scattered micro-calcifications throughout the former intima and media and extensive neovascularization. Macro-calcifications were less commonly observed, and predominantly associated with (remodeled) necrotic cores from (previous) atherosclerotic lesions.

Extensive neovascularization with vessels mainly originating from the medio-adventitial border^25^ and extending up into the ILT has been attributed to wall hypoxia caused by the luminal thrombus.^26^^,^^27^ Despite the attractiveness of this hypothesis, it could be argued that direct oxygen exchange between the aortic wall and the blood passing through the aorta is minimal due to absent erythrocyte-endothelium interaction^28^ and the high aortic flow,^29^ and that the aortic wall oxygen supply essentially relies on the vasa vasora network.^30^ Alternatively, it has been speculated that the abundant neovascularization in AAA disease may relate to abundance of proangiogenic signals such as HIF1a,^31^ VEGF,^32^ and CXCL8.^33^

Common, albeit not universal, observations for AAA were adventitial adipogenic degeneration, a process thought to reflect dysregulated mesenchymal cell differentiation^13^ and remodeling of the intraluminal thrombus. Although adhering thrombus is grossly removed during surgery, remnants of adhering thrombus were present in the majority of the samples. Movat stainings revealed different grades of remodeling, with ingrowth of the wall (mesenchymal cell influx, matrix deposition, and angiogenesis) in the abluminal thrombus in a subset of samples.

More variable aspects included the presence of scattered atherosclerotic lesions (see above), and presence of lymphocytic infiltrates and tertiary lymphoid-like structures. Lymphocytic infiltrates, diffusely distributed throughout all vascular wall layers, mainly consisted of T-cells and generally presented in close proximity of microvessels. Tertiary lymphoid-like structures presented as a garland of follicles located at the former medial-adventitial border zone. These structures were dominated by B- and T-cells, and associated with CXCL13 expression, a chemokine critically involved in organizing B-cell follicles and germinal center formation.^34^ Similar to tertiary lymphoid-like structures observed in atherosclerosis,^22^ full lymphoid maturation was missing: most lymphoid structures lacked the characteristic germinal centers (H&E staining), and lymphoid B-cells were all negative for the maturation marker CD21. Moreover, it was observed that the plasma cells located non-classically in the peripheral niches of the tertiary lymphoid-like structures rather than central in the germinal center core.^35^

Although systematic application of this first AAA classification system indicates a number of universal features for AAA, considerable variation was observed for other aspects (eg, inflammation, atherosclerotic lesions, and ILT organization). This apparent biological variation has potential implications for AAA research, with the need for robust group sizes in order to adequately cover the biological variability. The value of the scoring system is illustrated by two cases from the biobank that did not align with the classification.

### Limitations

The scheme is a first approach to generate a general multiparameter histopathological classification system for AAA disease. As such, the classification system may not fully encompass all aspects of the AAA disease spectrum. For example, due to the absence of protocolized size estimations and an accompanying large measurement variability,^36^ we refrained from including growth data as parameter. A further limitation is the lack of wall samples of AAA smaller than 50 mm. Also, the interpretation of atherosclerotic lesions in AAA sections is hampered by the shielding of the ILT, which may interfere with the natural history of atherosclerotic disease progression (such as interference with the intima-luminal blood interaction). Wall samples in this analysis were randomly chosen from an existing tissue bank. No stratification for specific factors such as age, sex, or comorbidities was performed. Although associations were found between the histological classification system components and clinical parameters, the power of this study was too low for regression analysis. As such, it is unclear whether the observed associations are covariants or causative. Finally, the proposed classification system should be considered as a “work in progress,” with room for future adaptations and/or extensions such as extension to smaller AAA and/or inclusion of aspects of the ILT, an aspect that could not be fully addressed in this study.

## Author Contributions

Conception and design: LB, JH, JL

Analysis and interpretation: LB, JC, JG, GJ, RL, LM, DW, BX, DY, JL

Data collection: LB, CG, CR, PV, JL

Writing the article: LB, JL

Critical revision of the article: LB, CG, JC, JG, JH, GJ, RL, LM, CR, PV, DW, BX, DY, JL

Final approval of the article: LB, CG, JC, JG, JH, GJ, RL, LM, CR, PV, DW, BX, DY, JL

Statistical analysis: LB, CG

Obtained funding: Not applicable

Overall responsibility: JL
