# Supplementary material for: A histopathological classification scheme for abdominal aortic aneurysm disease
Source: JVS Vasc Sci. 2021 Oct 7;2:260–73. doi: 10.1016/j.jvssci.2021.09.001 (PMC8605212; doi:10.1016/j.jvssci.2021.09.001)
Supplement: Supplemental material [file mmc1.docx]

**SUPPLEMENTAL MATERIAL**

**SUPPLEMENTAL PROTOCOLS**

**Supplemental protocol 1: Protocols for (immuno)histological stainings**

***H&E Staining:***

Deparaffinize and rehydrate slides. Rinse in demi water 5’. Haematoxylin 4’. Rinse in tap water and demi water. Eosin 1’. Rinse thoroughly in demi water. Dehydrate slides and clear in xylene, three to four changes. Mount with Pertex.

***Movat Pentachrome Staining:***

**Working solutions:**

1. 1% Alcian Blue Solution: 1 g Alcian Blue 8 GX (Merck, Burlington, US), 100 ml distilled water, 1 ml Glacial Acetic Acid (Sigma Aldrich, Saint Louis, US)
2. Alkaline Alcohol solution: 10 ml Ammonium Hydroxide (Merck, Burlington, US), 90 ml Ethanol 100%.
3. Elastic Hematoxylin Solution: 25 ml 10% Alcoholic Hematoxylin **(J)**, 25 ml Ethanol 100%, 25 ml 10% Ferric Chloride **(D)**, 25 ml Verhoeff’s Iodine Solution **(K)**.
4. 10% Ferric Chloride Solution: 10 g Ferric Chloride (Sigma Aldrich, Saint Louis, US), 100 ml distilled water
5. 5% Sodium Thiosulfate Solution: 5g Sodium Thiosulfate (Sigma Aldrich, Saint Louis, US), 100 ml distilled water
6. Biebrich Scarlet/Acid Fuchsin solution: pre-made from ScyTek Laboratories (Logan, United States).
7. 1% Acetic Acid Solution: 1 ml Glacial Acetic Acid, 99 ml distilled water
8. 5% Aqueous Phosphotungstic Acid solution: 5 g Phosphotungstic Acid (Sigma Aldrich, Saint Louis, US), 100 ml distilled water
9. 4% Alcoholic Saffron Solution: 4 g Saffron (Safranor Safran du Gâtinais, Échilleuses, France), 100 ml Ethanol 100%.
10. 10% Alcoholic Hematoxylin Solution: 10 g Hematoxylin (Merck, Burlington, US), 100 ml Ethanol 100%
11. Verhoeff’s Iodine Solution: 2 g Iodine Crystals (Sigma Aldrich, Saint Louis, US), 4 g Potassium Iodide (Sigma Aldrich, Saint Louis, US), 100 ml distilled water

**Protocol:**

1. Deparaffinization and rehydration of slides. 2. Rinse slides in distilled water. 3. Stain in 2 changes with **(A)** , both times for 15-25 minutes. 4. Rinse slides in running warm to hot water until clear. 5. Place slides in **(B)** for 30 minutes, then rinse in running tap water. 6. Stain in **(C)** for 20 minutes. 7. Rinse in running warm tap water. 8. Differentiate in 2% aqueous **(D)** for 5 seconds-2 minutes. 9. Place slides in **(E)** for about 1 minute. 10. Wash in running tap water and rinse in distilled water. 11. Stain in **(F)** for 1-1.5 minutes. 12. Rinse in distilled water. 13. Rinse in **(G)** for 7-12 seconds. 14. Place slides in **(H)** for 7-12 minutes. 15. Rinse in distilled water. 16. Rinse in **(G)** for 8-10 seconds. 17. Place in 2 changes of Ethanol 100%. 18. Stain in **(I)** for 1.5 minute and quickly rinse in Ethanol 100%. 19. Dehydration of slides. Mount with Pertex.

***Alizarin Red staining:***

Deparaffinize and rehydrate slides. Place in 1% Alizarin Red S solution 5’. Rinse in demi water. Dehydrate slides and clear in xylene, three to four changes. Mount with Pertex.

***IHC staining:***

**Protocol:**

Antibodies (see **Supplemental Table I** below) were diluted in PBS-1% albumin and incubated overnight at 4˚C. Heat-induced antigen retrieval was performed for 10 minutes at 95˚C and endogenous peroxidase activity blocked with hydrogen peroxide.

Anti-mouse EnVision+-HRP (Dako, Amstelveen, Holland) and donkey-anti-goat-HRP (AB97120; Abcam, Cambridge, UK) were applied as secondary antibodies and peroxidase activity was visualized using DAB (Dako, Amstelveen, Holland).

Nuclei were counter stained by Mayer’s hematoxylin (Merck Millipore, Holland).

**SUPPLEMENTAL TABLES**

**Supplemental Table I: Primary antibodies used in this study**

| **Antibody, clone/cat no** | **Host isotype (subclass)** | **Target** | **Antigen retrieval method (Tris-EDTA pH 9.2/ Citrate pH 6)** | **Dilution** | **Source** |
| --- | --- | --- | --- | --- | --- |
| Apo-B100, ab7616 | Goat IgG | Apolipoprotein B | Tris-EDTA | 1:4000 | Abcam |
| CD4, SC7219 | Rabbit IgG | T-helper cells | Tris-EDTA | 1:800 | Santa Cruz Biotechnology |
| CD8, C8/144B | Mouse IgG1 | Cytotoxic T-cells | Tris-EDTA | 1:200 | Dako |
| CD20, L26 | Mouse IgG2a | Pan B cell | Tris-EDTA | 1:4000 | Dako |
| CD21, 2G9 | Mouse IgG2a | Mature B cells, Follicular Dendritic Cells | Tris-EDTA | 1:50 | Thermofisher |
| CD68, KP1 | Mouse IgG1 | Macrophages and foam cells | Tris-EDTA | 1:6000 | Dako |
| CD138, MI15 | Mouse IgG1 | Plasma cells | Citrate | 1:400 | Dako |
| CXCL13, AF801 | Goat IgG | Lymphorganogenic chemokine | Citrate | 1:50 | R&D |
| Glycophorin-A, JC159 | Mouse IgG | Erythrocyte membranes | Citrate | 1:400 | Abcam |
| Mouse IgG (negative control for CD68) | - | - | Tris-EDTA | 1:6000 | Dako |
| Myeloperoxidase, A0398 | Rabbit IgG | Neutrophils | Tris-EDTA | 1:6000 | Dako |
| Tryptase, AA1 | Mouse IgG1 | Mast cells | Tris-EDTA | 1:5000 | Dako |

| ***Pt nr heatmap*** | ***Sex*** | ***Age (years)*** | ***Diameter AAA (mm)*** | ***Smoking status*** | ***Statin use*** | ***Anti hypertension medication use*** | ***Diabetes*** | ***History of cerebrovascular accident*** | ***History of coronary***  ***atherosclerosis*** | ***Atrial fibrillation*** | ***Peripheral artery disease*** | ***COPD*** | ***Chronic inflammatory disease*** | ***Genetic disease/fistula*** | ***Date of tissue harvest*** | ***Date of paraffin embedding*** |
| --- | --- | --- | --- | --- | --- | --- | --- | --- | --- | --- | --- | --- | --- | --- | --- | --- |
| 1 | M | 80 | 78 | Current | Yes | Yes | No | No | Yes | No | No | Unknown | No | No | 5-4-04 | 10-4-04 |
| 2 | M | 78 | 56 | Former | Yes | No | No | No | No | No | No | Unknown | Yes* | No | 6-6-05 | 15-6-05 |
| 3 | F | 70 | 50 | Current | Yes | No | No | No | No | No | Unknown | No | Yes† | No | 4-7-08 | 8-7-08 |
| 4 | M | 69 | 61 | Never | No | Yes | No | No | No | No | No | Unknown | No | No | 5-4-04 | 10-4-04 |
| 5 | M | 78 | 73 | Current | Yes | Yes | No | Yes | Yes | No | Yes | Yes | No | No | 12-11-10 | 23-11-10 |
| 6 | M | 77 | 74 | Former | No | Yes | No | Yes | No | No | Unknown | Yes | No | No | 7-11-11 | 17-11-11 |
| 7 | M | 70 | 74 | Former | No | No | No | No | Yes | Yes | No | No | No | No | 22-6-07 | 26-6-07 |
| 8 | M | 57 | 54 | Current | Yes | Yes | No | No | No | No | Unknown | No | No | No | 7-6-11 | 14-6-11 |
| 9 | F | 72 | 61 | Current | No | Yes | No | Yes | No | No | Unknown | Unknown | No | No | 29-1-03 | 6-2-06 |
| 10 | F | 70 | 53 | Former | Yes | Yes | No | No | Yes | No | Yes | No | Yes ‡ | No | 20-5-10 | 25-5-10 |
| 11 | M | 59 | 56 | Former | Yes | Yes | No | No | Yes | No | Unknown | Yes | No | No | 13-4-09 | 19-4-09 |
| 12 | M | 72 | 70 | Former | Yes | Yes | No | No | Yes | No | No | Unknown | No | No | 15-02-12 | 25-02-12 |
| 13 | M | 68 | 62 | Former | Yes | Yes | No | No | Yes | Yes | Unknown | Yes | No | No | 9-10-12 | 18-10-12 |
| 14 | M | 74 | 74 | Never | Yes | Yes | No | No | No | No | No | Unknown | No | No | 1-4-02 | 6-4-02 |
| 15 | F | 62 | 51 | Current | Yes | Yes | No | No | No | No | No | No | No | No | 6-6-05 | 15-6-05 |
| 16 | M | 63 | 89 | Former | No | Yes | No | No | Yes | No | No | Unknown | No | No | 16-3-07 | 27-3-07 |
| 17 | M | 69 | 55 | Former | Yes | Yes | Yes | No | No | No | No | Unknown | No | No | 1-4-11 | 11-4-11 |
| 18 | M | 77 | 72 | Former | Yes | Yes | No | Yes | No | No | No | Unknown | No | No | 14-4-09 | 20-4-09 |
| 19 | M | 66 | 63 | Current | Yes | Yes | No | No | No | No | Unknown | No | No | No | 6-6-11 | 15-6-11 |
| 20 | F | 82 | 70 | Never | No | Yes | No | No | No | No | Yes | Unknown | No | No | 5-6-08 | 10-6-08 |
| 21 | M | 70 | 62 | Current | No | Yes | No | No | Yes | Yes | No | Unknown | No | No | 5-12-06 | 16-12-06 |
| 22 | M | 67 | 56 | Current | No | No | No | Yes | No | No | No | Yes | No | No | 21-5-07 | 26-5-07 |
| 23 | M | 69 | 61 | Current | Yes | Yes | Yes | Yes | Yes | Yes | Yes | Yes | No | No | 2-7-10 | 9-7-10 |
| 24 | M | 69 | 56 | No | Yes | Yes | No | No | Yes | Yes | Unknown | No | Yes ‡ | No | 12-11-10 | 23-11-10 |
| 25 | M | 69 | 80 | Current | Yes | Yes | Yes | No | Yes | No | Yes | Yes | No | No | 18-5-04 | 29-5-04 |
| 26 | M | 68 | 54 | Current | No | Yes | No | No | Yes | No | Yes | Unknown | No | No | 14-5-08 | 20-5-08 |
| 27 | M | 68 | 55 | Former | No | Yes | No | No | Yes | Yes | No | Unknown | No | No | 21-3-05 | 27-3-05 |
| 28 | M | 68 | 50 | Current | Yes | Yes | No | No | Yes | No | No | Unknown | No | No | 01-6-09 | 11-6-09 |
| 29 | F | 65 | 68 | Former | Yes | Yes | No | Yes | No | No | Unknown | Unknown | Yes† | No | 7-6-05 | 15-6-05 |
| 30 | F | 67 | 77 | Current | Yes | Yes | No | No | Yes | No | No | Unknown | No | No | 12-6-09 | 22-6-09 |
| 31 | F | 69 | 50 | Former | Yes | No | No | No | No | No | Unknown | No | No | No | 22-8-12 | 30-8-12 |
| 32 | M | 74 | 100 | Former | Yes | Yes | No | No | No | No | No | Unknown | No | No | 21-6-07 | 30-6-07 |
| 33 | M | 81 | 44 | Never | No | No | Yes | No | Yes | No | Yes | Unknown | Yes § | No | 8-8-02 | 13-8-02 |
| 34 | M | 75 | 61 | Former | Yes | Yes | No | No | No | No | Yes | No | No | No | 10-11-03 | 17-11-03 |
| 35 | M | 71 | 70 | Former | No | No | Yes | Yes | No | No | Unknown | Unknown | No | No | 7-7-04 | 19-7-04 |
| 36 | M | 75 | 71 | Former | Yes | Yes | Yes | No | No | No | No | Unknown | No | No | 3-5-04 | 13-5-04 |
| 37 | M | 69 | 46 | Current | No | Yes | No | No | Yes | No | Yes | Yes | No | No | 9-9-11 | 15-9-11 |
| 38 | M | 67 | 51 | Current | No | No | No | Yes | No | No | Unknown | Yes | No | No | 19-3-07 | 25-3-07 |
| 39 | M | 69 | 65 | Current | No | No | No | No | No | No | Unknown | Yes | No | No | 23-1-06 | 30-1-06 |
| 40 | F | 68 | 49 | Current | Yes | Yes | No | No | No | No | Yes | No | No | No | 8-5-09 | 18-5-09 |
| 41 | M | 77 | 74 | Current | Yes | Yes | No | No | Yes | No | Unknown | Yes | No | No | 2-4-10 | 9-4-10 |
| 42 | M | 63 | 55 | Current | Yes | Yes | No | Yes | No | No | Yes | Yes | No | No | 26-2-03 | 3-3-03 |
| 43 | M | 73 | 58 | Never | Yes | Yes | No | No | Yes | No | No | Unknown | Yes ¶ | No | 4-4-08 | 10-4-08 |
| 44 | F | 78 | 53 | Current | Yes | Yes | No | No | No | Yes | No | No | No | No | 17-8-04 | 29-8-04 |
| 45 | F | 87 | 83 | Never | No | Yes | No | No | No | Yes | No | Unknown | No | No | 25-2-05 | 1-3-05 |
| 46 | M | 64 | 70 | Never | No | Yes | No | No | No | No | No | No | No | No | 08-10-12 | 15-10-12 |
| 47 | M | 65 | 85 | Never | Yes | Yes | No | No | Yes | Yes | Yes | Unknown | No | No | 19-5-05 | 30-6-05 |
| 48 | M | 75 | 100 | Former | Yes | Yes | No | No | No | No | Unknown | Unknown | No | No | 13-04-08 | 20-4-08 |
| 49 | F | 64 | 54 | Former | Yes | Yes | No | No | No | No | Unknown | No | No | No | 5-3-08 | 19-3-08 |
| 50 | M | 72 | 65 | Current | Yes | Yes | No | No | Yes | Yes | No | Unknown | No | No | 8-9-07 | 17-9-07 |
| 51 | M | 64 | 67 | Current | Yes | Yes | No | No | No | No | No | Yes | No | No | 11-12-11 | 19-12-11 |
| 52 | M | 69 | 100 | Former | No | No | No | No | No | No | Unknown | Unknown | No | No | 4-7-09 | 8-7-09 |
| 53 | M | 66 | 80 | Former | Yes | Yes | No | No | Yes | Yes | Unknown | No | No | No | 18-9-11 | 26-9-11 |
| 54 | M | 76 | 53 | Never | No | No | No | No | Yes | No | No | Unknown | No | No | 1-9-04 | 10-9-04 |
| 55 | M | 76 | 90 | Current | Yes | Yes | Yes | No | No | No | Yes | Yes | No | No | 20-9-10 | 27-9-10 |
| 56 | F | 72 | 57 | Current | Yes | Yes | No | No | No | Yes | No | Unknown | No | No | 2-8-06 | 13-8-06 |
| 57 | M | 66 | 82 | Former | No | No | No | Yes | No | Yes | No | Unknown | No | No | 25-4-08 | 30-4-08 |
| 58 | F | 72 | 72 | Former | Yes | Yes | No | No | No | No | Yes | Yes | Yes # | No | 1-4-02 | 10-4-02 |
| 59 | M | 69 | 55 | Former | Yes | Yes | No | No | Yes | No | No | Unknown | No | No | 8-5-06 | 15-5-06 |
| 60 | M | 89 | 74 | Current | No | Yes | Yes | No | No | Yes | Unknown | Unknown | No | No | 3-3-05 | 10-3-05 |
| 61 | M | 74 | 90 | Current | Yes | Yes | No | No | Yes | No | Unknown | Unknown | No | No | 20-4-09 | 29-4-09 |
| 62 | M | 67 | 105 | Former | Yes | Yes | No | Yes | No | Yes | No | Yes | No | No | 29-1-03 | 5-2-03 |
| 63 | M | 84 | 70 | Former | Yes | Yes | No | No | Yes | No | Yes | Unknown | No | No | 14-5-03 | 28-5-03 |
| 64 | M | 77 | 61 | Never | Yes | Yes | No | Yes | Yes | No | Unknown | Unknown | No | No | 4-1-07 | 13-1-07 |
| 65 | M | 73 | 55 | Current | No | No | No | No | Yes | No | Unknown | Unknown | No | No | 7-5-04 | 19-5-04 |
| 66 | M | 66 | 121 | Current | Yes | Yes | No | Yes | Yes | Yes | No | Yes | No | Yes** | 12-3-06 | 18-3-06 |
| 67 | M | 55 | 140 | Former | Yes | Yes | No | No | Yes | No | Unknown | Unknown | No | No | 21-2-03 | 27-2-03 |
| 68 | M | 77 | 90 | Current | Yes | No | No | No | No | No | Yes | No | No | No | 3-8-07 | 10-8-07 |
| 69 | M | 77 | 94 | Former | Yes | Yes | No | Yes | Yes | No | Unknown | Unknown | No | No | 16-1-08 | 22-2-08 |
| 70 | M | 64 | 75 | Current | Yes | No | No | No | Yes | No | Unknown | No | No | No | 7-5-03 | 14-5-03 |
| **Excluded patient samples¶¶** | | | | | | | | | | | | | | |  |  |
|  | M | 71 | 47 | Current | Yes | Yes | No | Yes | No | No | Yes | Unknown | No | Suggestive | 11-4-07 | 20-4-07 |
|  | M | 59 | 83 | Never | No | Yes | No | No | No | No | No | Unknown | No | Marfan's disease | 16-9-06 | 24-9-06 |

**Supplemental Table II:** Extensive description of AAA patients characteristics. **Abbreviations:** **CVA** (Cerebrovascular Accident), **AF** (Atrial Fibrillation), **PAD** (Peripheral Artery Disease), **COPD** (Chronic Obstructive Pulmonary Disease). **Symbols**: ***** Lyme disease, † Hashimoto’s thyreoiditis, ‡ Rheumatoid arthritis,

§ Paget's disease, ¶ Sarcoidosis, # Kidney transplantation due to membranous glomerulonephritis, ** Aorto-caval fistula, ¶¶ Two cases were excluded from analysis, one Marfan patient and one with characteristics suggestive for a genetic AAA variant (multiple aortic branch vessel aneurysms and four second degree family members with an AAA).

| **Non-inflammatory aortic diseases consensus classification** | | **Commentary** |
| --- | --- | --- |
| **Component** | **Gradation^±^** | AAA |
| **1. Mucoid extracellular matrix (ECM) accumulation** | 0. Absent  1. Focal (≤10% of media involved)  2. Multifocal (multiple areas, 11–30% of media involved)  3. Extensive (>30% of media involved) | Absent in AAA. |
| **2. Elastic fiber alternations (fragmentation and/or loss, thinning and disorganization)** | 0. Absent  1. Focal  2. Multifocal  3. Extensive | All AAA samples in this study displayed (almost) full elastolysis. |
| **3. Laminar medial collapse** | Appearance: thin/dense | Laminar units are perished in AAA due to (almost) complete elastolysis. |
|  | 0. Absent  1. Focal  2. Multifocal  3. Extensive |  |
| **4. SMC disorganization** | 1. Focal  2. Multifocal  3. Extensive | (Very) rare process in AAA (n=2 in this study; see *Supplemental Figure 1.2*). A rather complete distorted organization of vessel wall in all AAA samples. |
| **5. Smooth muscle cell (SMC) nuclei loss** | Type: patchy/band-like | Virtually all AAA samples in this study were characterized by extensive SMC loss, some AAA samples were almost acellular. |
|  | 0. Absent  1. Rare  2. Frequent  3. Extensive |  |
|  |  |  |
|  |  |  |
| **6. Medial fibrosis** | 0. Absent  1. Focal  2. Multifocal  3. Extensive | All AAA samples in this study were characterized by extensive fibrosis. |
|  |  |  |
|  |  |  |

**Supplemental Table IIIa: *Evaluation of Consensus Classification for Histological Surgical Non-Inflammatory Aortic Sections.***

| **Inflammatory aortic diseases consensus classification** | | **Commentary** |
| --- | --- | --- |
| **Component** | **Gradation or inflammatory pattern subclassification** | AAA |
| **1. Atherosclerosis (with excessive inflammation)** | 0. No significant atherosclerosis  1. Mild atherosclerosis  2. Moderate atherosclerosis  3. Severe atherosclerosis  4. Atherosclerosis with plaque disruption and surface thrombus  5. Calcific atherosclerosis | Vast majority of AAA samples contained atherosclerotic lesions. Most severe lesions present were classified as necrotic cores. Clusters of mature FCP lesions were rare.  Distinct inflammatory footprints for aortic atherosclerosis and AAA were observed (*Supplemental Figure 1.3*). |
| **2. (Peri)aortitis** | **1. Granulomatous/giant cell pattern**: clusters of epithelioid macrophages with(out) giant cells or compact/well-formed granulomas.  **2. Lymphoplasmacytic pattern:** Lymphocytes and plasma cells without granulomatous component.  **3. Mixed inflammatory pattern:** All/most inflammatory cell types without an overt granulomatous pattern.  **4. Suppurative pattern:** neutrophilic abscesses with necrosis and cell debris. | Mixed inflammatory pattern in AAA. |

**Supplemental Table IIIb: *Evaluation of Non-inflammatory Consensus Classification for Histological Surgical Inflammatory Aortic Sections.***

**Supplemental Table IV:** Exploration of regional AAA heterogeneity in the longitudinal axis of four patients (1-4). Every letter represents a tissue block (e.g 1a is the most proximal tissue block of patient 1). We assessed the regional diversity along the longitudinal axis, since collection of circumferential AAA sections was not approved by the medical ethical review board.

| **Patient nr** | **Fibrosis in intima/media zone** | **Mesenchymal cell loss in intima/media zome** | **Inflammation-transmural lymphoid infiltration** | **Inflammation-lymhpoid follicles in adventitia** | **Atherosclerotic lesions** | **Wall thickness (intima+media)** | **Extent of neovessel formation** | **Intraluminal thrombus organization** | **Calcification** | **Elastic fibre degradation** | **Adventitial adipogenic degeneration** |
| --- | --- | --- | --- | --- | --- | --- | --- | --- | --- | --- | --- |
| **1a** |  |  |  |  |  |  |  |  |  |  |  |
| **1b** |  |  |  |  |  |  |  |  |  |  |  |
| **1c** |  |  |  |  |  |  |  |  |  |  |  |
| **1d** |  |  |  |  |  |  |  |  |  |  |  |
| **1e** |  |  |  |  |  |  |  |  |  |  |  |
| **1f** |  |  |  |  |  |  |  |  |  |  |  |
| **1g** |  |  |  |  |  |  |  |  |  |  |  |
| **1h** |  |  |  |  |  |  |  |  |  |  |  |
| **2a** |  |  |  |  |  |  |  |  |  |  |  |
| **2b** |  |  |  |  |  |  |  |  |  |  |  |
| **2c** |  |  |  |  |  |  |  |  |  |  |  |
| **2d** |  |  |  |  |  |  |  |  |  |  |  |
| **2e** |  |  |  |  |  |  |  |  |  |  |  |
| **2f** |  |  |  |  |  |  |  |  |  |  |  |
| **2g** |  |  |  |  |  |  |  |  |  |  |  |
| **2h** |  |  |  |  |  |  |  |  |  |  |  |
| **2i** |  |  |  |  |  |  |  |  |  |  |  |
| **2j** |  |  |  |  |  |  |  |  |  |  |  |
| **3a** |  |  |  |  |  |  |  |  |  |  |  |
| **3b** |  |  |  |  |  |  |  |  |  |  |  |
| **3c** |  |  |  |  |  |  |  |  |  |  |  |
| **3d** |  |  |  |  |  |  |  |  |  |  |  |
| **3e** |  |  |  |  |  |  |  |  |  |  |  |
| **3f** |  |  |  |  |  |  |  |  |  |  |  |
| **4a** |  |  |  |  |  |  |  |  |  |  |  |
| **4b** |  |  |  |  |  |  |  |  |  |  |  |
| **4c** |  |  |  |  |  |  |  |  |  |  |  |
| **4d** |  |  |  |  |  |  |  |  |  |  |  |
| **4e** |  |  |  |  |  |  |  |  |  |  |  |
| **4f** |  |  |  |  |  |  |  |  |  |  |  |
| **4g** |  |  |  |  |  |  |  |  |  |  |  |
| **4h** |  |  |  |  |  |  |  |  |  |  |  |
| **4i** |  |  |  |  |  |  |  |  |  |  |  |

**Supplemental Table V:** Table of Pearson Correlation tests between histological-clinical parameters and histological-histological parameters. Significant associations (*p<.05) are in **bold**.

|  |  | Sex | Age | Indication | Diameter AAA | Smoking status | Statin use | Antihypertensive medication use | Diabetes | History of cerebrovascular accident | History of coronary atherosclerosis | Atrial fibrillation | Peripheral Artery Disease | COPD | Aortic wall thickness | Intimal/medial fibrosis | Mesenchymal cell loss | Inflammation-transmural lymphoid infiltrates | Inflammation- TLO-like structures in adventitia | Atherosclerotic lesions | Neovascularization | Intraluminal thrombus organization |
| --- | --- | --- | --- | --- | --- | --- | --- | --- | --- | --- | --- | --- | --- | --- | --- | --- | --- | --- | --- | --- | --- | --- |
| Mesenchymal cell loss |  | -.06 | -.05 | -.10 | -.03 | .12 | .02 | -.13 | .07 | .19 | .00 | -.04 | .22 | -.18 | -.14 | **.24*** | X | .18 | **.36*** | -.13 | .13 | -.03 |
| Intimal/medial fibrosis |  | -.12 | .10 | -.18 | -.20 | -.04 | -.01 | -.13 | .14 | .15 | .18 | -.16 | .17 | .06 | -.08 | X | **.24*** | -.22 | -.09 | .06 | .13 | -.21 |
| Aortic wall thickness |  | -.21 | -.15 | .16 | **.40*** | .21 | -.04 | -.05 | -.20 | .03 | .01 | -.02 | .19 | .06 | X | -.08 | -.14 | .13 | .21 | **.41*** | -.01 | .01 |
| Inflammation-transmural lymphoid infiltrates |  | -.08 | **-.43*** | -.23 | -.02 | **.25*** | .23 | **.29*** | -.07 | .00 | .07 | .02 | .02 | -.11 | .13 | -.22 | .18 | X | **.30*** | .03 | .14 | **.24*** |
| Inflammation- TLO-like structures in adventitia |  | -.13 | -.14 | .10 | .06 | .23 | **.28*** | -.07 | -.01 | .17 | -.11 | -.02 | -.01 | -.07 | .21 | -.09 | **.36*** | **.30*** | X | -.03 | .11 | **.31*** |
| Neovascularization |  | -.10 | -.08 | .11 | .13 | -.02 | .06 | .18 | -.21 | .07 | .16 | .10 | .04 | -.10 | -.01 | .13 | .13 | .14 | .11 | **-.24*** | X | -.11 |
| Atherosclerotic lesions |  | -.04 | .03 | .08 | .14 | .16 | **-.24*** | **-.27*** | -.07 | .02 | -.13 | -.21 | .03 | .21 | **.41*** | .06 | -.13 | .03 | -.03 | X | **-.24*** | .10 |
| Intraluminal thrombus organization |  | -.06 | **-.29*** | -.06 | -.02 | **.26*** | .15 | -.04 | -.04 | -.08 | -.16 | .04 | -.09 | -.07 | .01 | -.21 | -.03 | **.24*** | **.31*** | .13 | -.11 | X |

**SUPPLEMENTAL FIGURES**


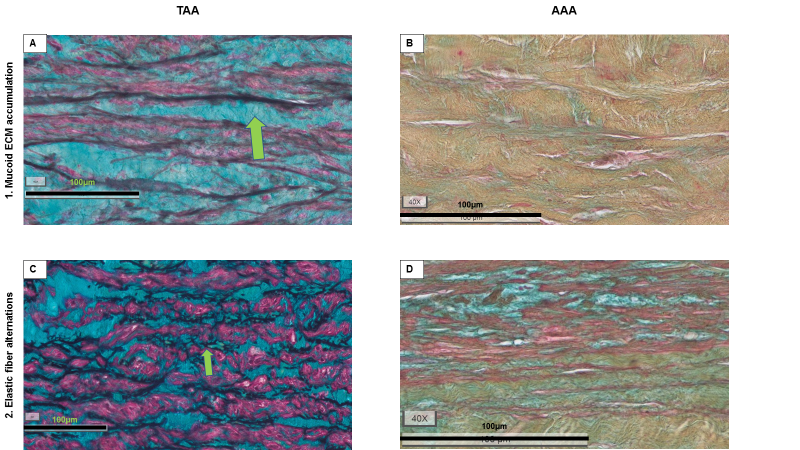


**
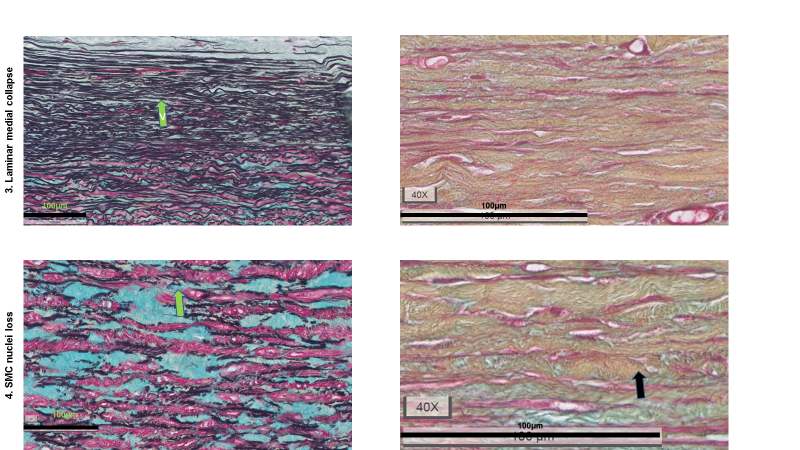
**

**
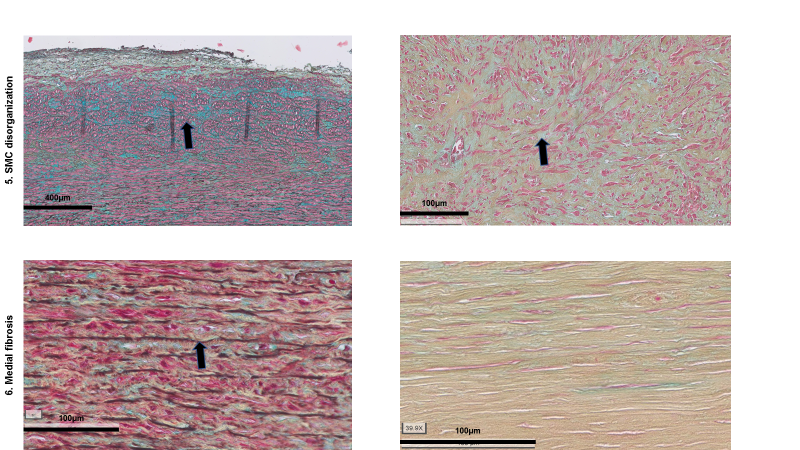
**

**Supplemental Figure 1.1: *Application of the Consensus classification for non-inflammatory ascending aortic pathology on AAA disease illustrates the contrasts between TAA and AAA.***

Movat Pentachrome stainings of TAA and AAA, color legend: *blue*: proteoglycans, *yellow*: collagen, *green*: colocalization of proteoglycans and collagen, *black*: elastin, *red*: smooth muscle cells (SMC) and fibrinogen, *purple*: nuclei. (**A, arrow**) Mucoid (highly hydrophilic glycosaminoglycans) ECM (extracellular matrix) accumulation, encountered in TAA, is absent in AAA (**B**). While in TAA elastic fiber fragmentation is seen (**C, arrow**), AAA is characterized by (almost) complete elastolysis (**D**). Laminar collapse as seen in TAA (**E, arrow**) is therefore absent in AAA due to full elastolysis (**F**). SMC disorganization, a rare process in TAA that is primarily associated with genetic aortopathies (**G**), was also very rare in AAA (**H**; see *Supplemental Figure 1.2* for more details). SMC nuclei loss in AAA (**J**) is more extreme than in TAA (**I**). Medial fibrosis (collagenous rich, mesenchymal poor and elastin poor matrix, reflected in the Movat staining by a ochre yellow cell matrix) in AAA (**L**) is far more outspoken than in TAA (**K**).


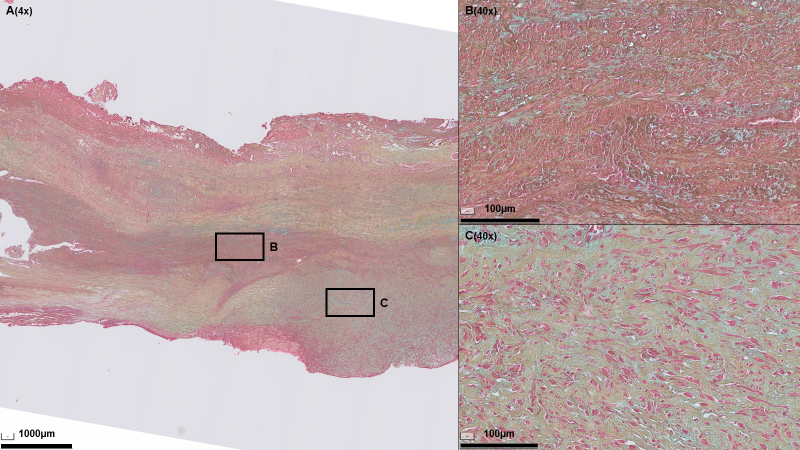


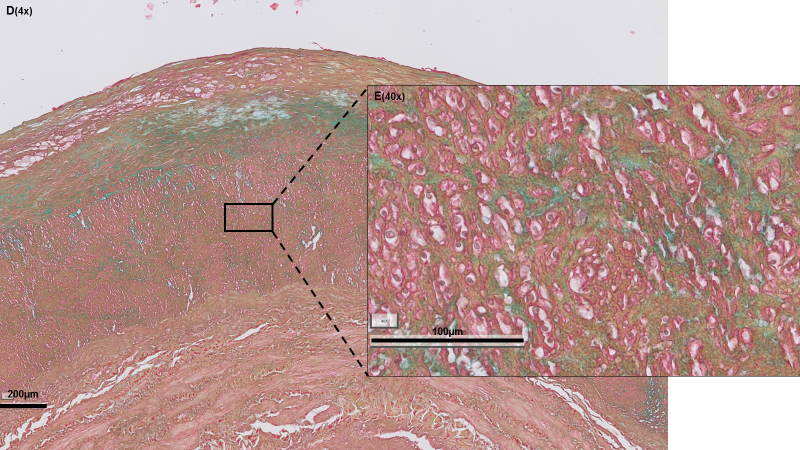


**Supplemental Figure 1.2: *Atypical smooth muscle cell (SMC) organization observed in two AAA sections.*** For legend of Movat Pentachrome staining see Supplemental Figure 1.1. (**A**) Retrospectively (after histological analysis), this AAA section that did not align with the classification system was of a patient with Marfan syndrome with a thoracic aortic aneurysm as well. Note the extensive SMC disorganization (**B**/**C**). The other aberrant section (**D**), also characterized by SMC disorganization (**E**) was of a AAA patient that presented with multiple aortic branch vessel aneurysms (iliac, femoral, popliteal) and had four family members with AAA disease, which may be in the context of a genetic aortic disease as well.

*
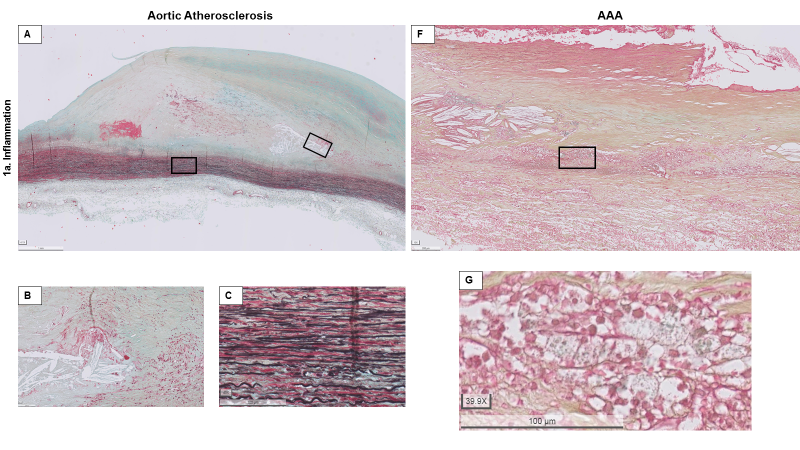
*

*
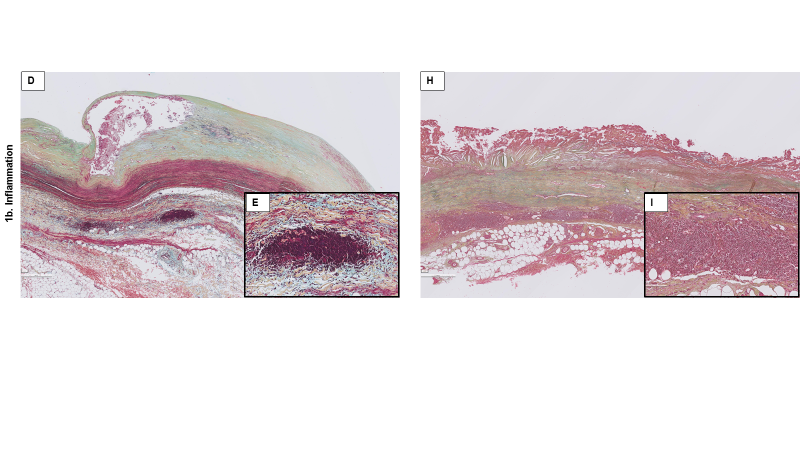
*

**Supplemental Figure 1.3: *Evaluation of the Consensus classification for inflammatory aortic pathology on AAA disease illustrates a distinct inflammatory footprint for aortic atherosclerosis and AAA disease.***

Movat Pentachrome stainings of aortic atherosclerosis and AAA. For color legend, see Supplemental Figure 1.1. (**A**) Inflammation in progressive aortic atherosclerosis (Late Fibroatheroma (LFA)) is clearly associated with the intimal lesion (viz. the shoulder regions of the cap, covering a necrotic core, and in direct vicinity of the necrotic core (**B**). The relatively intact media in LFA, although compressed directly under the large necrotic core (**C**), illustrates that the atherosclerotic process is mainly confined to the intima. Lymphoid follicles are absent in atherosclerosis, with the exception of the Plaque Rupture (not shown) and Thin-cap fibroatheromas (**D**), in which lymphoid follicles are located in the adventitia directly under the necrotic core (**E**).

In contrast, in AAA, the structure of the intima as well as of the media is extensively destructed due to fibrotic changes in the ECM (**F;** collagenous rich, mesenchymal poor and elastin poor matrix, reflected in the Movat staining by a ochre yellow cell matrix). A further contrast is seen for inflammation: in AAA inflammatory infiltrates are transmural (in all vessel layers) and diffuse (**G**), and/or concentrated in close relationship to the adventitial plexus as a garland of (tertiary-like) lymphoid follicles (**H/I**).

**Supplemental Figure 2.1-2.9: (Patho)histomorphological aspects** **of AAA disease**

**
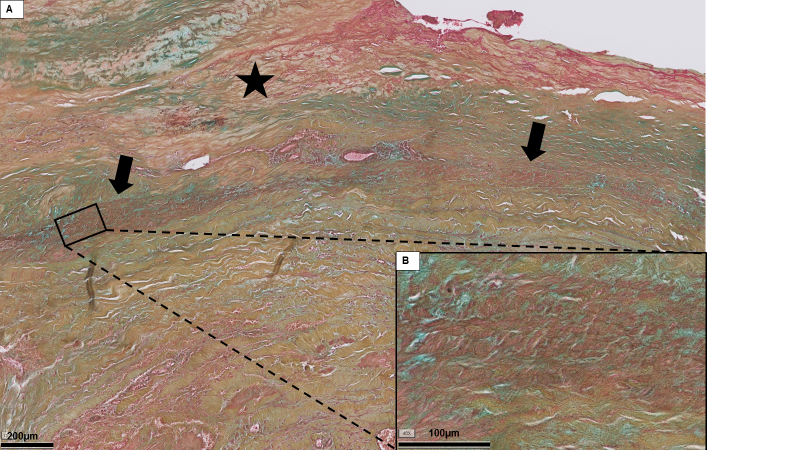
**

**
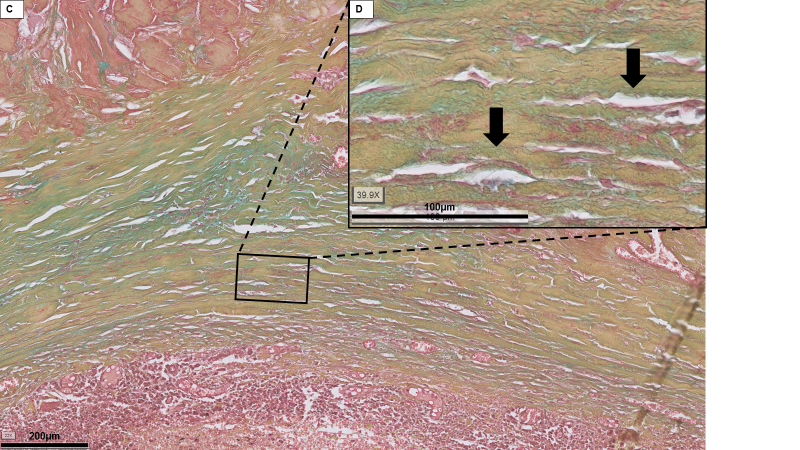
**

**Supplemental Figure 2.1:** Movat Pentachrome staining of two AAA sections. Color legend: *blue*: proteoglycans, *yellow*: collagen, *green*: colocalization of proteoglycans and collagen, *black*: elastin, *red*: smooth muscle cells (SMC) and fibrinogen, *purple*: nuclei. **Mesenchymal cell loss** was either characterized by a patchy pattern (**A**; **arrows** indicate patches of mesenchymal cell-rich areas (**B**) and the **star** indicates a mesenchymal cell depleted area) or a diffuse pattern (**C**), in which the mesenchymal cell loss is quite uniformly distributed in the vessel wall (**D**; arrows indicate mesenchymal cells (elongated)).


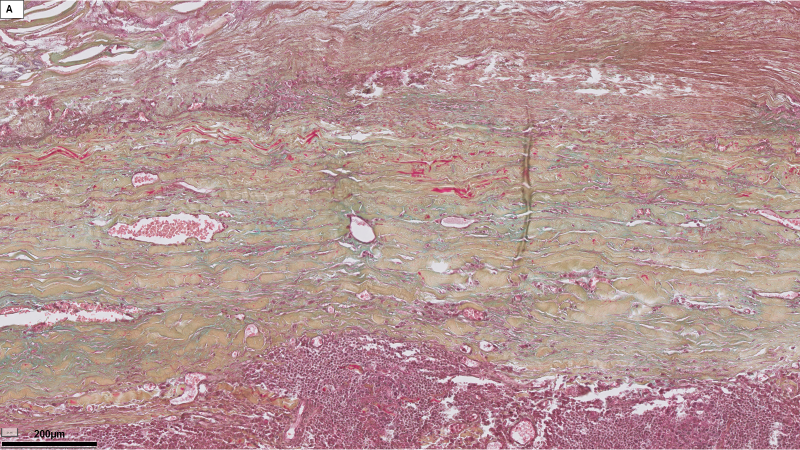


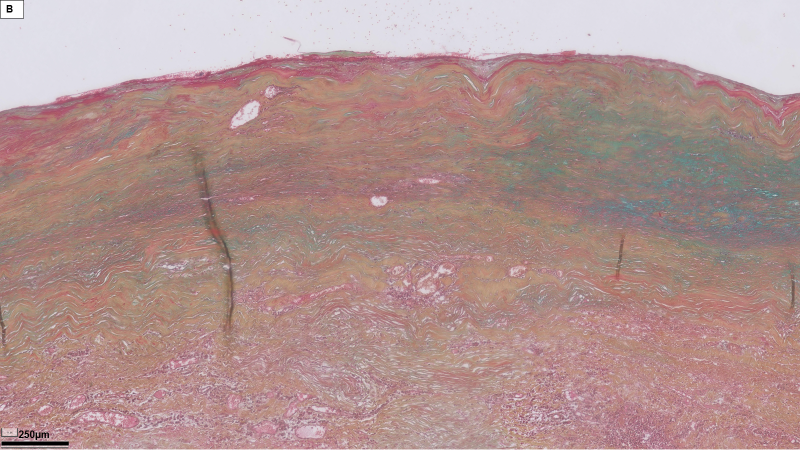


**Supplemental Figure 2.2:**. For color legend see Supplemental Figure 5.1. **Fibrosis**, defined as an collagenous rich, mesenchymal poor and elastin poor matrix, reflected in the Movat staining by an ochre yellow cell matrix; destructed all landmarks of the inimal/medial border. Fibrosis was semi-quantitatively graded in the former intima/media zone (see Supplemental Figure 2.4 for a description more in detail). Moreover, intimal/medial fibrosis was either characterized by a patchy pattern (**A**) or a diffuse pattern (**B**).


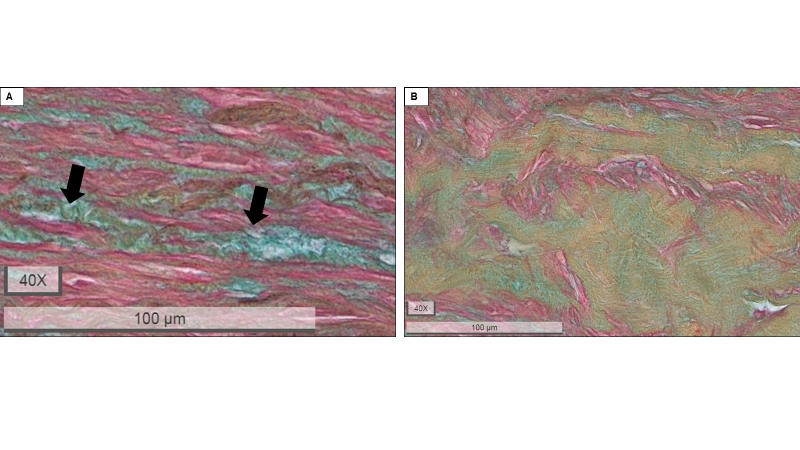


**Supplemental Figure 2.3:** Near complete (98%) **elastolysis** with patchy elastic fiber remnants (A; arrows indicate small elastic fiber remnants) versus full elastin loss in AAA (**B**). For color legend see Supplemental Figure 2.1.


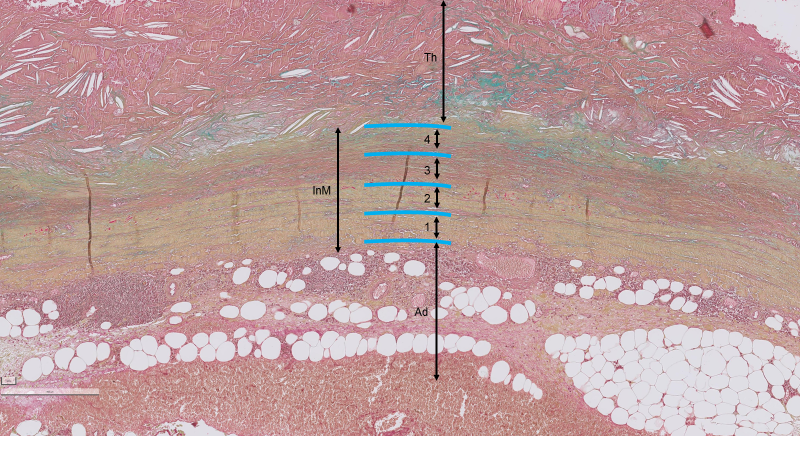


**Supplemental Figure 2.4: Illustrations of the landmarks for the intima/media zone thickness (InM)** was measured as the largest distance from the luminal margin of the intima up to the characteristic circumferential boundary zone in AAA. This zone is characterized by the presence of a vascular plexus and lymphoid follicles. This reference zone coincides with the medio-adventitial border in reference aorta.

The luminal thrombus (**Th**) was not considered. Adventitia (**Ad**) was also excluded in this measurement, because of the ambiguous boundary zones, and secondary tissue loss as result of the tissue preparation and sample processing.

Since demarcations between the intima and media were completely obliterated, an alternative approach was applied to describe anatomical locations: the intima-media zone was parted in 4 equal zones (**1-4**).

**
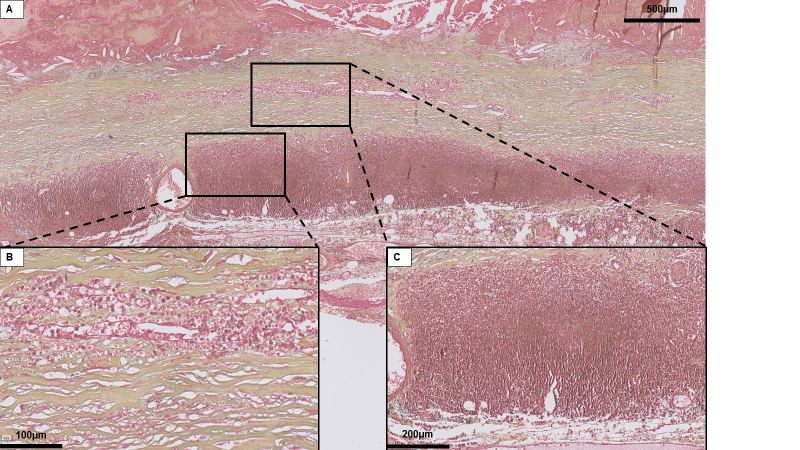
**

**
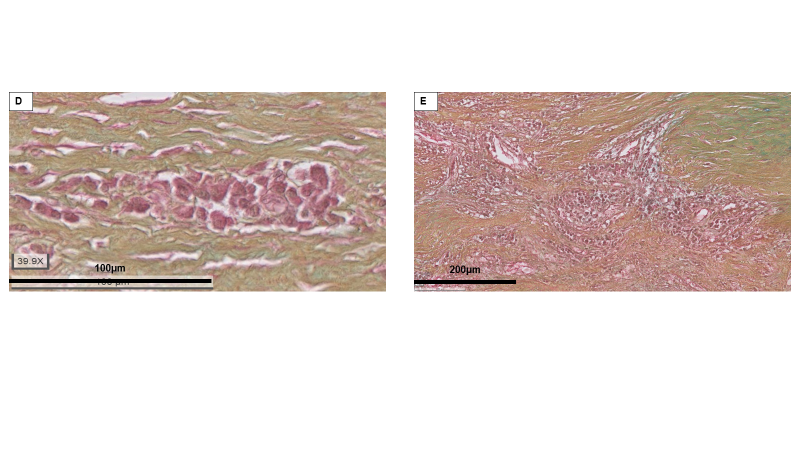

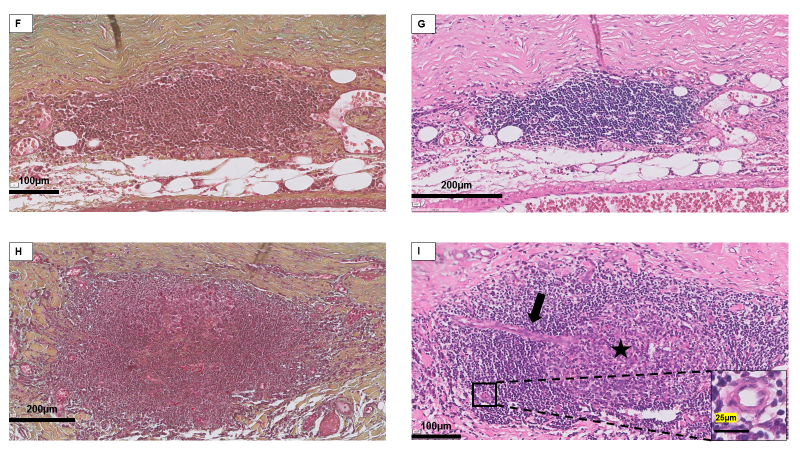
**

**Supplemental Figure 2.5: Clusters of lymphoid cells** in AAA (**A;** Movat) were characterized by two non-exclusive patterns: perivascular, transmural unorganized lymphoid infiltrates (**B;** Movat); and garland-like aggregation of lymphoid follicles, situated around the adventitial vessel plexus (**C**; Movat). The unorganized transmural infiltrates were scored as small (**D**; defined as <50 cells; Movat) or large (**E**; ≥50 cells; Movat). The development of tertiary-like structures were either considered early (**F**, Movat**; G**, H&E), defined as: unencapsulated lymphoid follicles with organization visible (reticular fibres), but without germinal centre; or late (**H**, Movat**; I**, H&E): organized unencapsulated lymphoid follicles with a germinal centre. Germinal centre morphology is characterized by diffusely organized lymphocytes surrounded by densely packed lymphocytes, which is most clear on H&E stainings (**I**, **star**). Moreover, high endothelial venules, that are venules which are lined by characteristic endothelial cells with a plum, cuboidal morphology (I, zoom box); or lymphatic vessels (**I**, **arrow**) were considered a feature of late TLO-structures. For color legend of Movat stainings see Supplemental Figure 2.1.

**
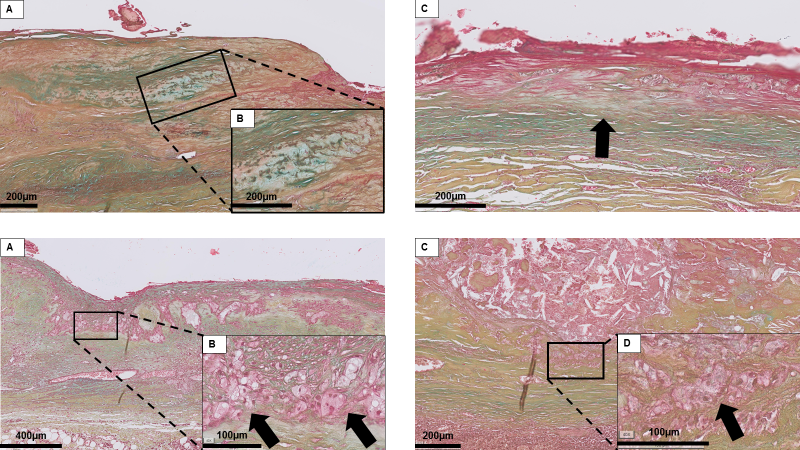
**

**
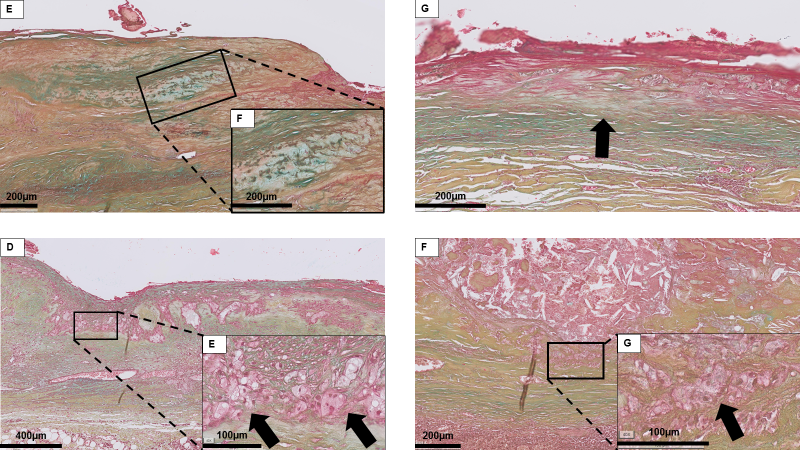
**

**
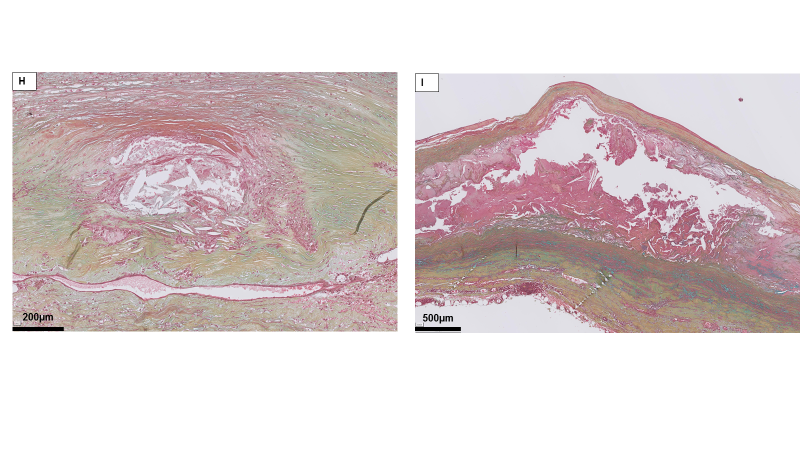
**

**
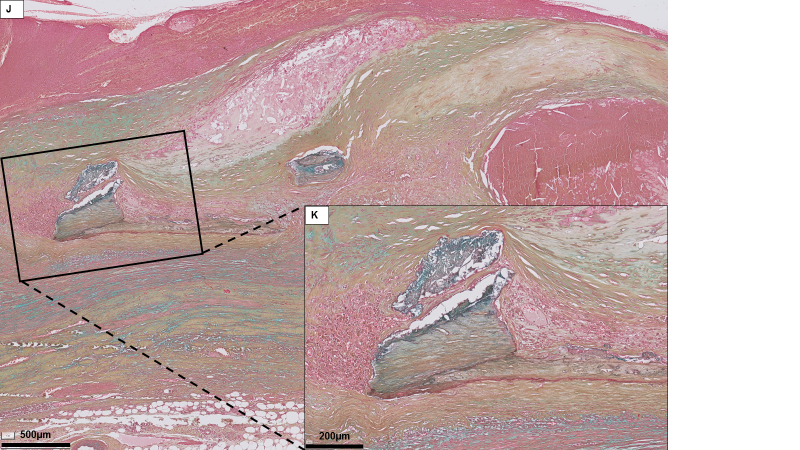
**

**Supplemental Figure 2.6: Atherosclerotic lesions in AAA**, evaluated by Movat stainings. An early atherosclerotic lesion is the accumulation of foam cells, that in AAA was either located in the former intima/media (**A**/**B**), or directly located under the intraluminal thrombus (**C**/**D**). They transition into Lipid pools (**E**/**F**), that are proteoglycans and hyaluronan rich (light blue on Movat) extracellular lipid accumulations. These lipid pools may be directly located under the intraluminal thrombus (**G**, **arrow**). Lipid-rich necrotic cores were either deeply located (**H**) or at the surface of the former intima (**I**). Calcified sheets (**J**/**K**) are a component of stabilized fibrocalcific plaques.

For color legend see Supplemental Figure 2.1.

**
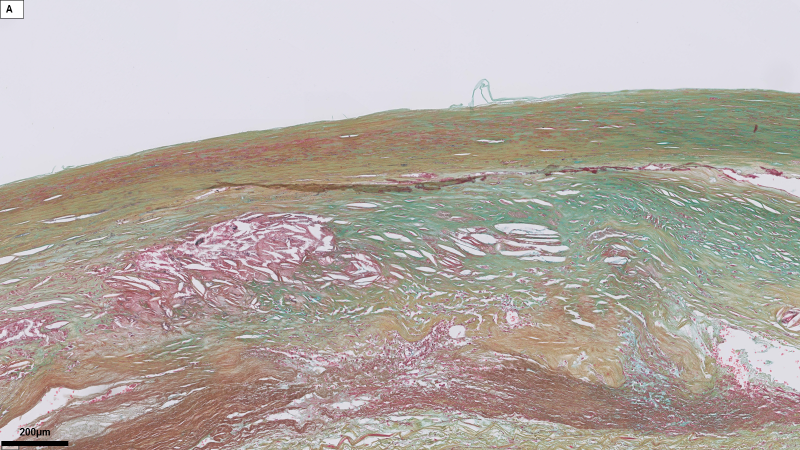
**

**
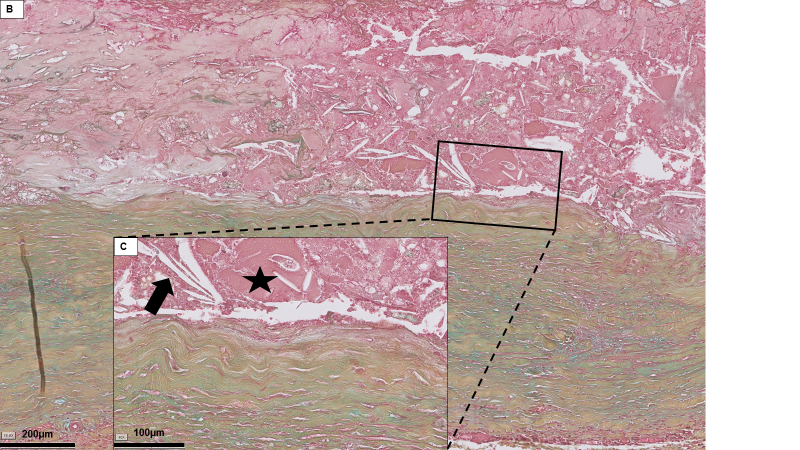
**

**
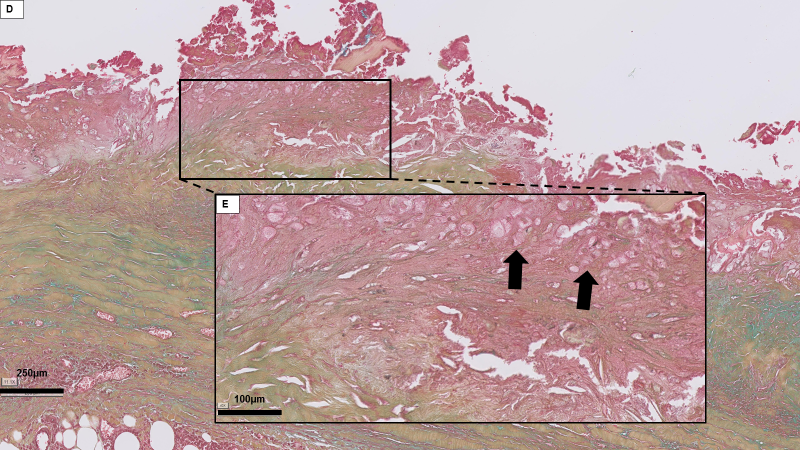
**

**
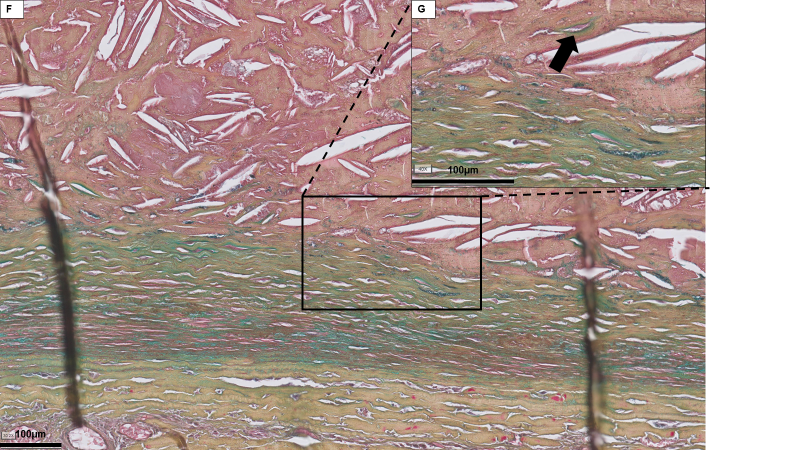
**

**
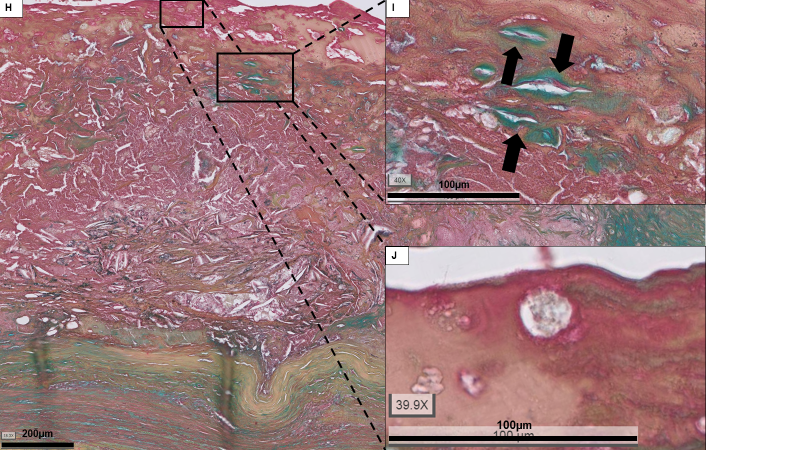
**

**Supplemental Figure 2.7: Intraluminal thrombus organization**, evaluated by Movat stainings. An intraluminal thrombus is present in the majority of AAA disease, however insert **A** shows an AAA specimen without an ILT. Insert **B** shows a relatively immature adhering ILT that is rich in fibrin (asterisk in **C**) and cholesterol (arrows in **C**; oblong spiked clefts). Various degrees of ILT reorganization were identified: reorganizing ILT, characterized by incorporation into the intima (**D**), without or with immune cell infiltration (**E**; arrows point to foam cells). Sings of an organized ILT were considered extracellular matrix deposition (**F**) and presence of spindle shaped cells in the ILT (**G**; arrow). Highly organized ILTs (**H**) were considered to be characterized by extracellular matrix deposition, spindle shaped cell ingrowth (**I**) and ingrowth of capillaries (**J**). For color legend see Supplemental Figure 2.1.


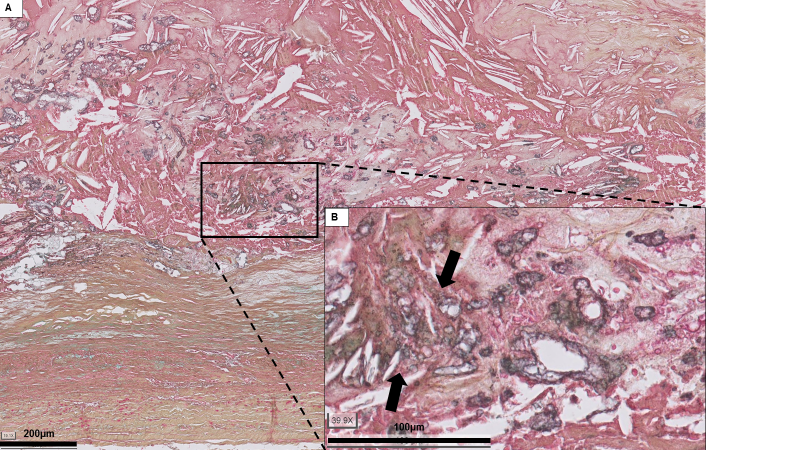


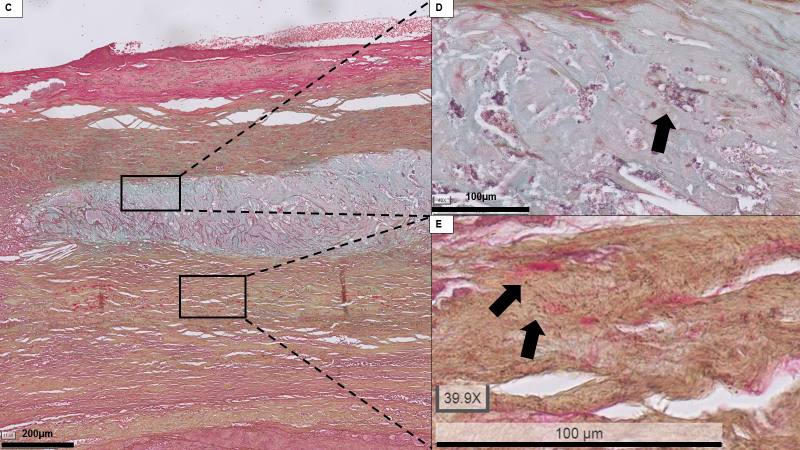


**Supplemental Figure 2.8: Microcalcifications in AAA**, evaluated by Movat stainings. Microcalcifications (*black* in Movat staining) in the aorta may be located in the intraluminal thrombus (**A**), where they are associated with cholesterol crystals (arrows in **B**; oblong spiked clefts). Progressive microcalcifications may also be positioned in the inner aortic wall (**D**; calcifications in lipid pool, recognizable by proteoglycan and hyaluronan rich extracellular matrix (light blue on Movat)) and/or the outer aortic wall (**E**; arrows point to microcalcifications).

For complete color legend see Supplemental Figure 2.1.

**
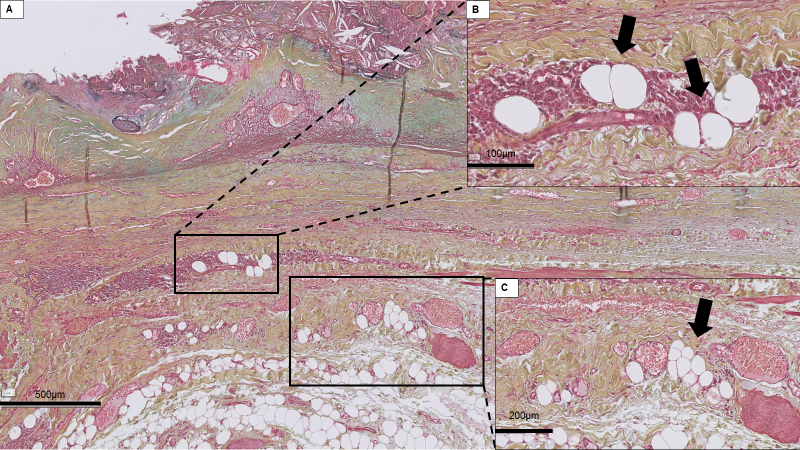
**

**Supplemental Figure 2.9: (A) Adventitial adipogenic degeneration**, shown by a Movat staining, is characterized by isolated adipocyte clusters (**B**, **arrows**) in the adventitia, without connection of the periaortic adipose tissue, and with intertwining strands or matrix or immune cell infiltrates in between the clusters (**C**, **arrows**).

For color legend see Supplemental Figure 2.1.

**Supplemental figure 3: Subtle morphological differences between AAA associated necrotic cores and classic aortic necrotic cores were observed, yet immunohistological stainings point to classic atherosclerotic etiology.**


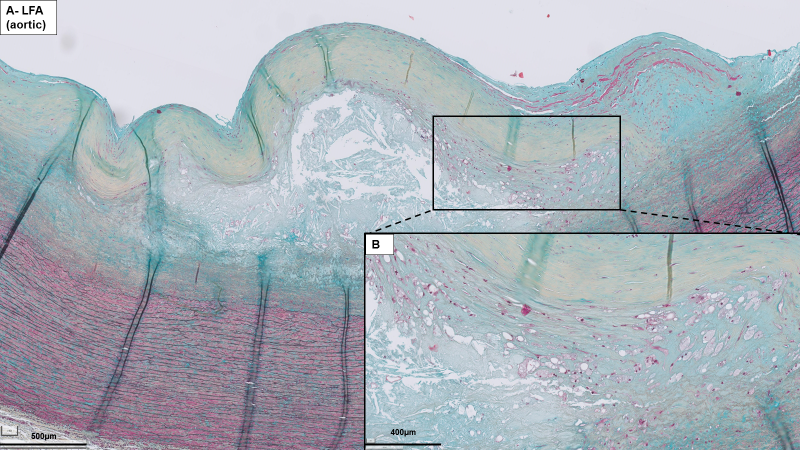


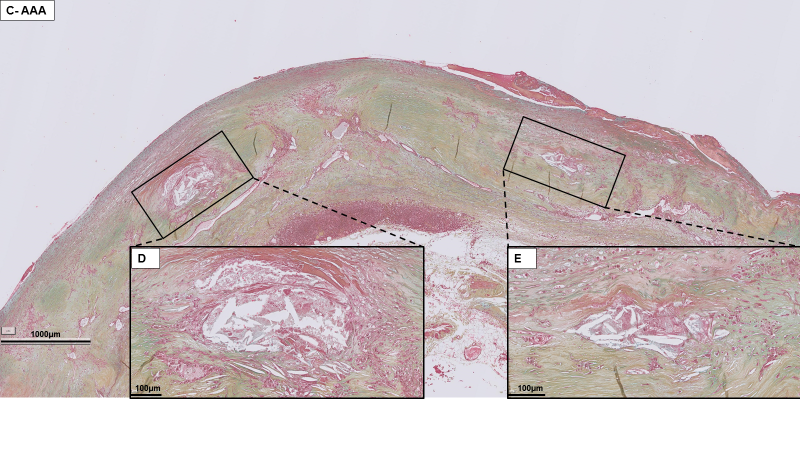


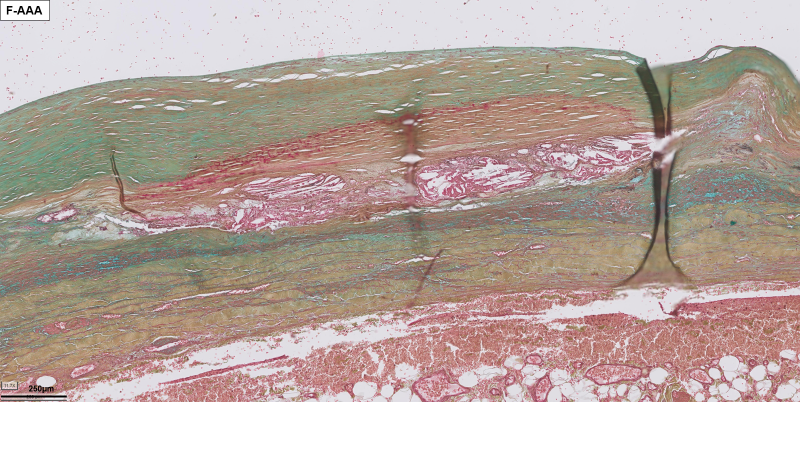
*Color legend to Movat stainings (****A****/****C****/****F****): blue: proteoglycans, yellow: collagen, green: colocalization of proteoglycans and collagen, black: elastin, red: smooth muscle cells and fibrinogen, purple: nuclei.*

**Supplemental Figure 3.1. Subtle contrasting morphological appearances** **between a subset of AAA-associated necrotic cores** (**C**/**F**) **and the classic infra-renal progressive atherosclerotic lesions** (**A**; Late Fibroatheroma (LFA)) were observed. In aortic atherosclerosis, the large necrotic core is situated in the intima with an underlying media and overlying fibrous cap with inflammation in the shoulder regions. Necrotic cores in AAA were generally smaller and better ‘structured’, and were more deeply located in the vascular wall. As a consequence, the classic fibrous cap with its inflammatory shoulders was generally missing.


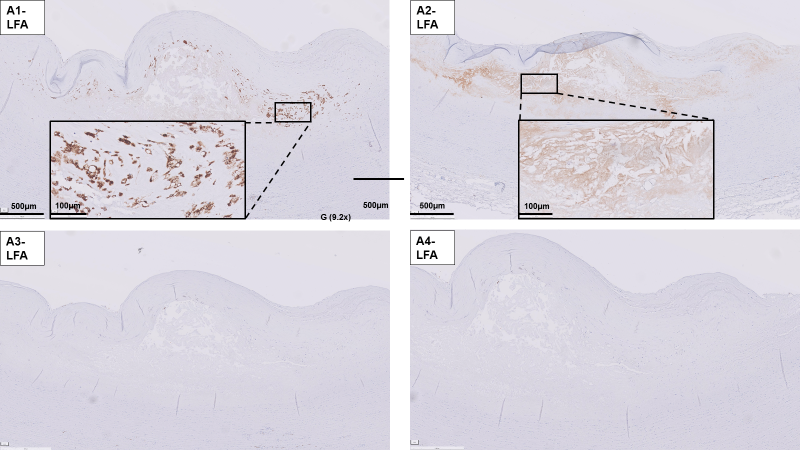


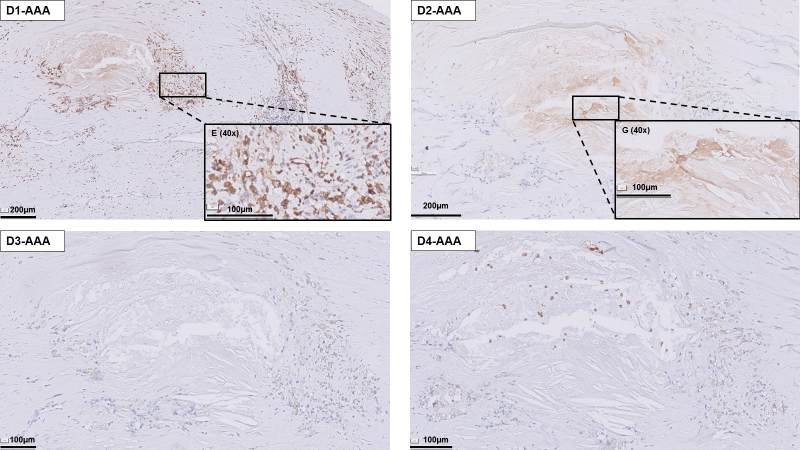


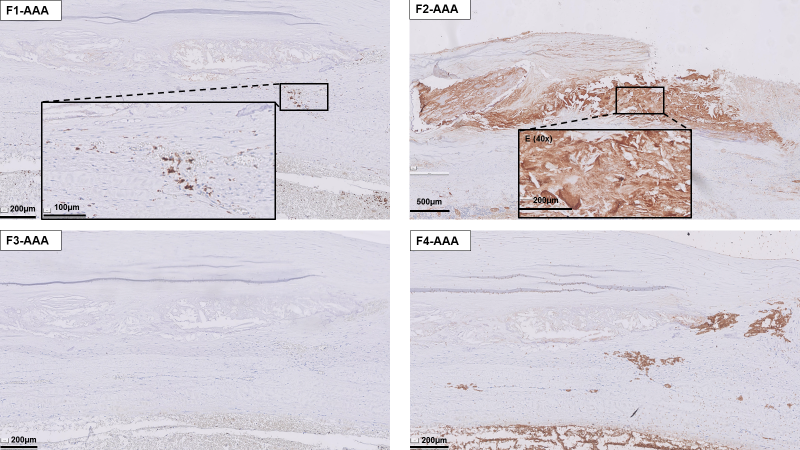


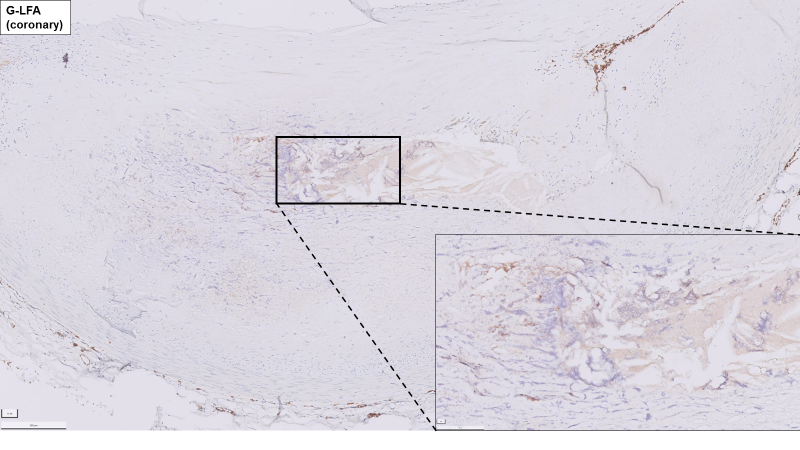


**Supplemental Figure 3.2: Identification of a classic atherogenic contributor in AAA associated necrotic cores.** IHC stainings **A1**-**A4** correspond to Movat Staining A, **D1**-**D4** correspond to Movat Staining D, et cetera.

To assess whether the origin of necrotic cores, next to morphology, in AAA were also different from classic necrotic cores in aortic atherosclerosis**,** it was reasoned that the atherosclerotic lesions in AAA disease may originate from two non-exclusive routes: infiltration and retention of lipoproteins as seen in classical aortic atherosclerosis (Apolipoprotein B100+ core (**A2**), infiltrated by CD68+ lipid loaden macrophages (**A1**); and alternatively, through accumulations of cholesterol derived from erythrocyte membranes (Glycophorin-A+) trapped in the thrombus^19^. The relevance of this phenomenon is clearly illustrated by the isolated cholesterol crystals in the inner intimal aspect of the intraluminal thrombus (Supplemental Figure 2.6f).

Similar to the necrotic cores in the reference atherosclerotic aorta, necrotic cores in AAA were infiltrated by macrophages and foam cells (**D1**, and to a lesser extent in **F1**) and were positive for ApoB100 (**D2**/**F2**). This concurs with a classic atherosclerotic etiology. The absence of Glycophorin-A expression in the aortic atherosclerotic lesions(**A4**/**D4**/**F4**) reflects that plaque haemorrhages do not contribute to the majority of aortic atherosclerotic lesions, in contrast to coronary atherosclerotic lesions (**G**).

Panels (**A3**/**D3**/**F3**) show a negative control for CD68 (Mouse IgG in same dilution as CD68 antibody).


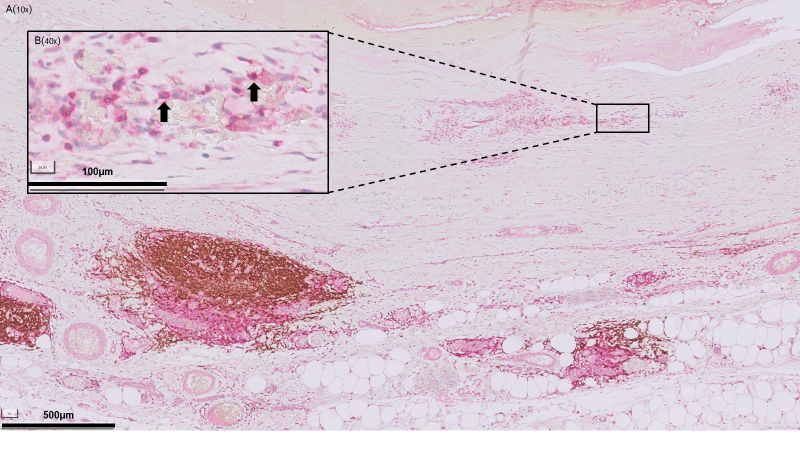


**Supplemental Figure 4.1:** **T-cell and B-cell distribution.** While T-cells (in *red*) were present in lymphoid infiltrates in the former intima-media zone as well as in the adventitia, B-cells (in *brown*) were primarily present in lymphoid follicles in the medio-adventitial border.

**
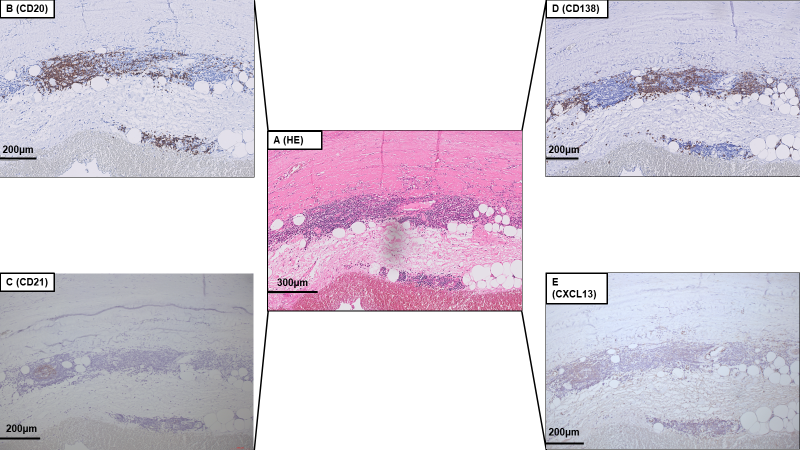
**

**
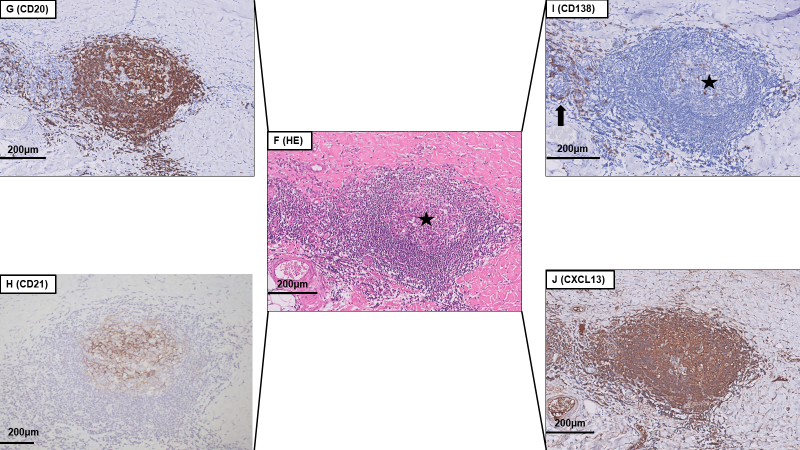
**

**
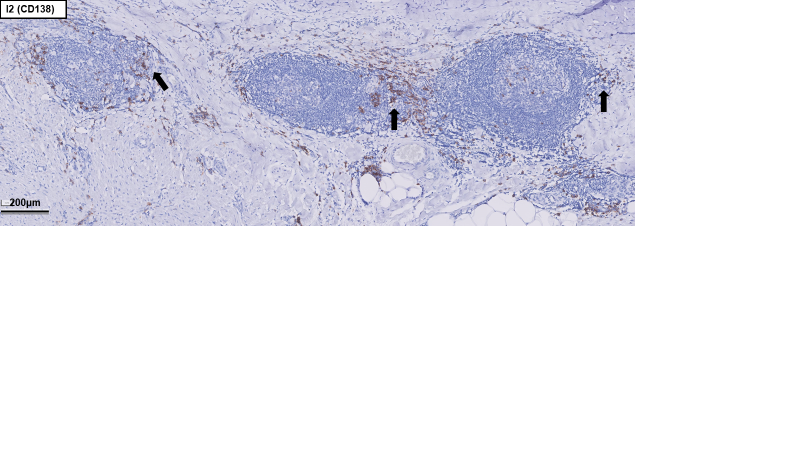
**

**Supplemental Figure 4.2:** **Indications for lack of B cell maturation in Tertiary lymphoid organ (TLO)-like structures.** Two adventitial tertiary lymphoid organ-like structures with various maturation grades (early in **A-E** (**A** (HE Staining): no germinal centre) and late in **F-J** (**F** (HE Staining): germinal centre)) were consecutively stained for CD20, CD21, CXCL13 and CD138. B-cells (CD20+) in AAA sections are primarily located within TLO-like structures in the medio-adventitial border (see also *Supplemental Figure 4.1*). Indeed a strong expression of CXCL-13, a B-cell lympho-organic cytokine, was found in both early and late TLO-like structures. Note that plasma cells (CD138+) mainly reside in peripheral niches (**D**/**I**, **I2** for I in context of adventitia). Meshworks of follicular dendritic cells are positive for CD21 (**C**/**H**), while B-cells in both early and late TLO-like structures are grossly negative for CD21 (**C**/**H**). These results indicate that although signals promoting follicle assembly are present in TLO-like structures, critical signals for B cell maturation are lacking.

**Supplemental Figure 5: Illustration of contribution of adaptive immune system in AAA disease: presence of macrophages, neutrophils and mast cells**

**
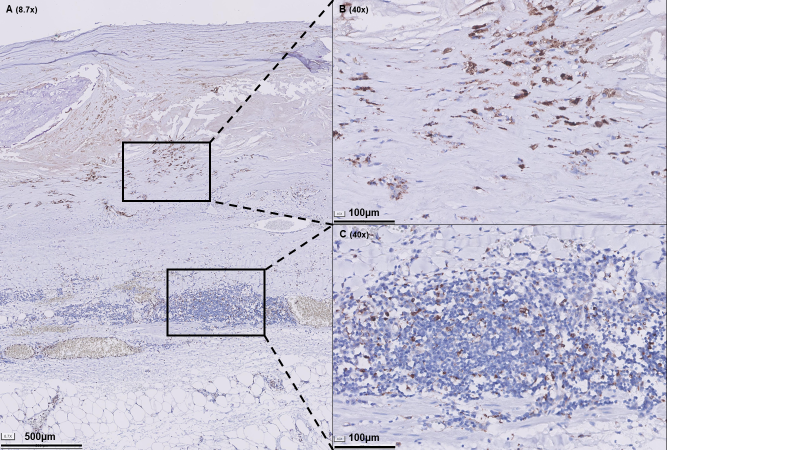
**

**Supplemental Figure 5.1: *Representative image of macrophage (CD68+) distribution in AAA.*** Macrophage infiltration was observed throughout all vessel layers (**A**). In atherosclerotic lesions in AAA, macrophage infiltrated in the necrotic cores (**B**). Macrophages were also present in TLO-like structures (**C**).

**
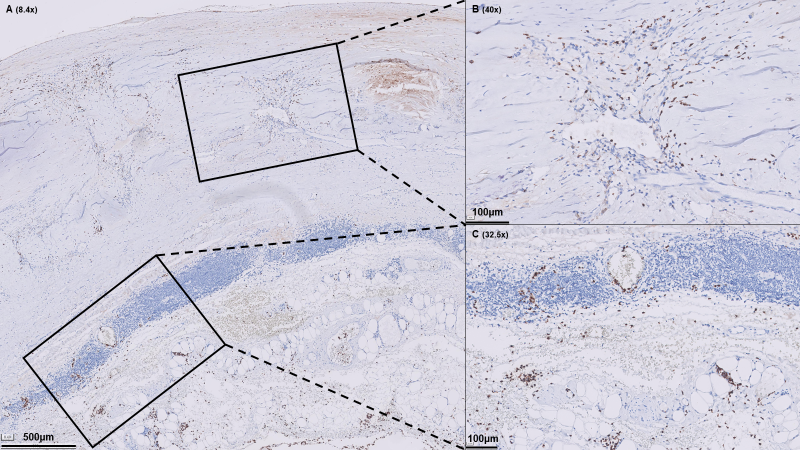
**

**Supplemental Figure 5.2: *Representative image of neutrophil (Myeloperoxidase+) distribution in AAA.*** Neutrophil infiltration was diffusely present throughout all vessel layers (**A**). In the intima and media, neutrophils primarily associated with microvessels (**B**). In the adventitia, neutrophils were present in close proximity to TLO-like structures (**C**), but in other regions of the adventitia prominent neutrophil infiltration was seen as well (**C**).

**
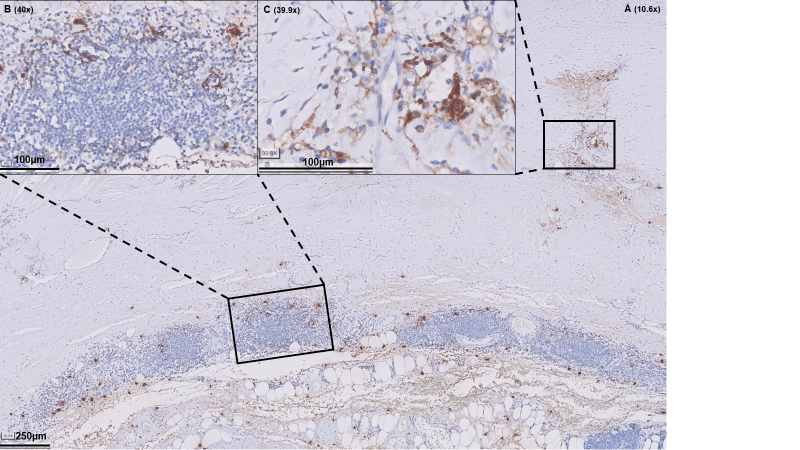
**

**Supplemental Figure 5.3: *Representative image of mast cell (Tryptase+) distribution in AAA.*** Mast cells infiltrated diffusely in all vessel layers. In the intima and media, infiltrates primarily associated with microvessels (**C**). In the adventitia, mast cells were present in close proximity to TLO-like structures (**B**), but solitary mast cells were also present.

**Supplemental Figure 6: Movat staining is suitable for evaluation of calcifications in decalcified AAA specimens**


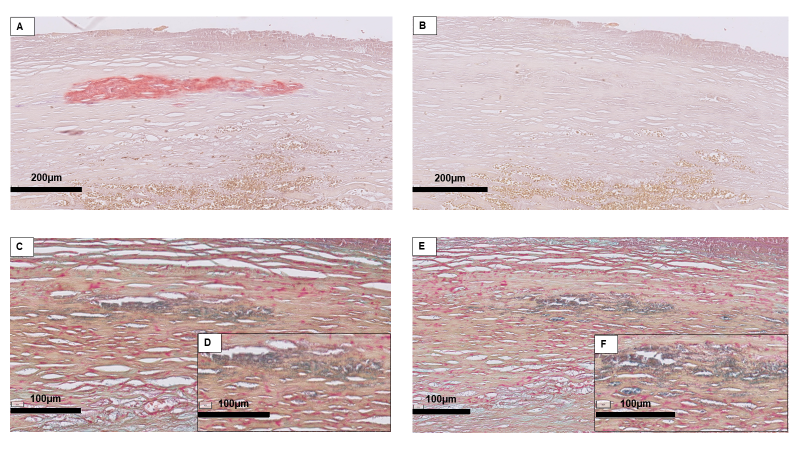
 For color legend of Movat Stainings see **Supplemental Figure 3.1**. (**A**) Alzarin Red S staining (calcium staining) of an AAA section that was not decalcified prior to sectioning; calcium deposits are visible in bright red. Panel **B** shows a consecutive section of the same AAA specimen as in **A**, but decalcified (60 minutes in Kristensen’s) after sectioning. Thus, decalcification clearly eliminates calcium deposits that are identified by Alzarin Red S staining.

In contrast, no apparent difference in calcium detection is observed in Movat stainings of consecutive sections of A and B that were respectively not decalcified (**C**/**D**) and sections that were decalcified after sectioning (**E**/**F**). This indicates that Movat stainings identify (remnant) structures that are associated with calcium, rather than the calcium itself.
